# International comparisons of laboratory values from the 4CE collaborative to predict COVID-19 mortality

**DOI:** 10.1038/s41746-022-00601-0

**Published:** 2022-06-13

**Authors:** Griffin M. Weber, Chuan Hong, Zongqi Xia, Nathan P. Palmer, Paul Avillach, Sehi L’Yi, Mark S. Keller, Shawn N. Murphy, Alba Gutiérrez-Sacristán, Clara-Lea Bonzel, Arnaud Serret-Larmande, Antoine Neuraz, Gilbert S. Omenn, Shyam Visweswaran, Jeffrey G. Klann, Andrew M. South, Ne Hooi Will Loh, Mario Cannataro, Brett K. Beaulieu-Jones, Riccardo Bellazzi, Giuseppe Agapito, Mario Alessiani, Bruce J. Aronow, Douglas S. Bell, Vincent Benoit, Florence T. Bourgeois, Luca Chiovato, Kelly Cho, Arianna Dagliati, Scott L. DuVall, Noelia García Barrio, David A. Hanauer, Yuk-Lam Ho, John H. Holmes, Richard W. Issitt, Molei Liu, Yuan Luo, Kristine E. Lynch, Sarah E. Maidlow, Alberto Malovini, Kenneth D. Mandl, Chengsheng Mao, Michael E. Matheny, Jason H. Moore, Jeffrey S. Morris, Michele Morris, Danielle L. Mowery, Kee Yuan Ngiam, Lav P. Patel, Miguel Pedrera-Jimenez, Rachel B. Ramoni, Emily R. Schriver, Petra Schubert, Pablo Serrano Balazote, Anastasia Spiridou, Amelia L. M. Tan, Byorn W. L. Tan, Valentina Tibollo, Carlo Torti, Enrico M. Trecarichi, Xuan Wang, The Consortium for Clinical Characterization of COVID-19 by EHR (4CE), James R. Aaron, Adem Albayrak, Giuseppe Albi, Anna Alloni, Danilo F. Amendola, François Angoulvant, Li L. L. J. Anthony, Fatima Ashraf, Andrew Atz, Paul Avillach, Paula S. Azevedo, James Balshi, Brett K. Beaulieu-Jones, Antonio Bellasi, Vincent Benoit, Michele Beraghi, José Luis Bernal-Sobrino, Mélodie Bernaux, Romain Bey, Surbhi Bhatnagar, Alvar Blanco-Martínez, Martin Boeker, John Booth, Silvano Bosari, Robert L. Bradford, Gabriel A. Brat, Stéphane Bréant, Nicholas W. Brown, Raffaele Bruno, William A. Bryant, Mauro Bucalo, Emily Bucholz, Anita Burgun, Tianxi Cai, Aldo Carmona, Charlotte Caucheteux, Julien Champ, Krista Y. Chen, Jin Chen, Lorenzo Chiudinelli, Kelly Cho, James J. Cimino, Tiago K. Colicchio, Sylvie Cormont, Sébastien Cossin, Jean B. Craig, Juan Luis Cruz-Bermúdez, Jaime Cruz-Rojo, Mohamad Daniar, Christel Daniel, Priyam Das, Batsal Devkota, Audrey Dionne, Rui Duan, Julien Dubiel, Loic Esteve, Hossein Estiri, Shirley Fan, Robert W. Follett, Thomas Ganslandt, Noelia García-Barrio, Lana X. Garmire, Nils Gehlenborg, Emily J. Getzen, Alon Geva, Tobias Gradinger, Alexandre Gramfort, Romain Griffier, Nicolas Griffon, Olivier Grisel, Alba Gutiérrez-Sacristán, Larry Han, David A. Hanauer, Christian Haverkamp, Derek Y. Hazard, Bing He, Darren W. Henderson, Martin Hilka, Kenneth M. Huling, Meghan R. Hutch, Richard W. Issitt, Anne Sophie Jannot, Vianney Jouhet, Ramakanth Kavuluru, Chris J. Kennedy, Kate F. Kernan, Daniel A. Key, Katie Kirchoff, Jeffrey G. Klann, Isaac S. Kohane, Ian D. Krantz, Detlef Kraska, Ashok K. Krishnamurthy, Trang T. Le, Judith Leblanc, Guillaume Lemaitre, Leslie Lenert, Damien Leprovost, Molei Liu, Qi Long, Sara Lozano-Zahonero, Sadiqa Mahmood, Sarah E. Maidlow, Adeline Makoudjou, Anupama Maram, Patricia Martel, Marcelo R. Martins, Jayson S. Marwaha, Aaron J. Masino, Maria Mazzitelli, Arthur Mensch, Marianna Milano, Marcos F. Minicucci, Bertrand Moal, Taha Mohseni Ahooyi, Jason H. Moore, Cinta Moraleda, Jeffrey S. Morris, Karyn L. Moshal, Sajad Mousavi, Douglas A. Murad, Shawn N. Murphy, Thomas P. Naughton, Carlos Tadeu Breda Neto, Jane Newburger, Kee Yuan Ngiam, Wanjiku F. M. Njoroge, James B. Norman, Jihad Obeid, Marina P. Okoshi, Karen L. Olson, Gilbert S. Omenn, Nina Orlova, Brian D. Ostasiewski, Nathan P. Palmer, Nicolas Paris, Lav P. Patel, Miguel Pedrera-Jiménez, Ashley C. Pfaff, Emily R. Pfaff, Danielle Pillion, Sara Pizzimenti, Hans U. Prokosch, Robson A. Prudente, Andrea Prunotto, Víctor Quirós-González, Rachel B. Ramoni, Maryna Raskin, Siegbert Rieg, Gustavo Roig-Domínguez, Pablo Rojo, Paula Rubio-Mayo, Paolo Sacchi, Carlos Sáez, Elisa Salamanca, Malarkodi Jebathilagam Samayamuthu, L. Nelson Sanchez-Pinto, Arnaud Sandrin, Nandhini Santhanam, Janaina C. C. Santos, Fernando J. Sanz Vidorreta, Maria Savino, Juergen Schuettler, Luigia Scudeller, Neil J. Sebire, Pablo Serrano-Balazote, Patricia Serre, Arnaud Serret-Larmande, Mohsin Shah, Zahra Shakeri Hossein Abad, Domenick Silvio, Piotr Sliz, Jiyeon Son, Charles Sonday, Andrew M. South, Francesca Sperotto, Zachary H. Strasser, Amelia L. M. Tan, Bryce W. Q. Tan, Suzana E. Tanni, Deanne M. Taylor, Ana I. Terriza-Torres, Patric Tippmann, Emma M. S. Toh, Yi-Ju Tseng, Andrew K. Vallejos, Gael Varoquaux, Margaret E. Vella, Guillaume Verdy, Jill-Jênn Vie, Shyam Visweswaran, Michele Vitacca, Kavishwar B. Wagholikar, Lemuel R. Waitman, Demian Wassermann, Griffin M. Weber, Martin Wolkewitz, Scott Wong, Zongqi Xia, Xin Xiong, Ye Ye, Nadir Yehya, William Yuan, Alberto Zambelli, Harrison G. Zhang, Daniela Zöller, Valentina Zuccaro, Chiara Zucco, Isaac S. Kohane, Tianxi Cai, Gabriel A. Brat

**Affiliations:** 1 Department of Biomedical Informatics, Harvard Medical School, Boston, USA; 2 Department of Biostatistics and Bioinformatics, Duke University, Durham, USA; 3 Department of Neurology, University of Pittsburgh, Pittsburgh, USA; 4 Department of Neurology, Massachusetts General Hospital, Boston, USA; 5 Department of biomedical informatics, Hôpital Européen Georges Pompidou, Assistance Publique - Hôpitaux de Paris, Paris, France; 6 Department of biomedical informatics, Hôpital Necker-Enfants Malade, Assistance Publique Hôpitaux de Paris (APHP), University of Paris, Paris, France; 7 Department of Computational Medicine & Bioinformatics, Internal Medicine, Human Genetics, and School of Public Health, University of Michigan, Ann Arbor, USA; 8 Department of Biomedical Informatics, University of Pittsburgh, Pittsburgh, USA; 9 Department of Medicine, Massachusetts General Hospital, Boston, USA; 10 Department of Pediatrics-Section of Nephrology, Brenner Children’s Hospital, Wake Forest School of Medicine, Winston Salem, USA; 11 Department of Anaesthesia, National University Health System, Singapore, Singapore, Singapore; 12 Department of Medical and Surgical Sciences, Data Analytics Research Center, University Magna Graecia of Catanzaro, Italy, Catanzaro, Italy; 13 Department of Electrical, Computer and Biomedical Engineering, University of Pavia, Italy, Pavia, Italy; 14 Department of Legal, Economic and Social Sciences, University Magna Graecia of Catanzaro, Italy, Catanzaro, Italy; 15 Department of Surgery, ASST Pavia, Lombardia Region Health System, Pavia, Italy; 16 Departments of Biomedical Informatics, Pediatrics, Cincinnati Children’s Hospital Medical Center, University of Cincinnati, Cincinnati, USA; 17 Department of Medicine, David Geffen School of Medicine at UCLA, Los Angeles, USA; 18 IT department, Innovation & Data, APHP Greater Paris University Hospital, Paris, France; 19 Department of Pediatrics, Harvard Medical School, Boston, USA; 20 Unit of Internal Medicine and Endocrinology, Istituti Clinici Scientifici Maugeri SpA SB IRCCS, Pavia, Italy; 21 Massachusetts Veterans Epidemiology Research and Information Center (MAVERIC), VA Boston Healthcare System, Boston, USA; 22 Department of Electrical Computer and Biomedical Engineering, University of Pavia, Italy, Pavia, Italy; 23 VA Informatics and Computing Infrastructure, VA Salt Lake City Health Care System, Salt Lake City, USA; 24 Health Informatics, Hospital Universitario 12 de Octubre, Madrid, Spain; 25 Department of Learning Health Sciences, University of Michigan, Ann Arbor, USA; 26 Department of Biostatistics, Epidemiology, and Informatics, University of Pennsylvania Perelman School of Medicine, Philadelphia, USA; 27 Institute for Biomedical Informatics, University of Pennsylvania Perelman School of Medicine, Philadelphia, USA; 28 Digital Research, Informatics and Virtual Environments (DRIVE), Great Ormond Street Hospital for Children, UK, London, UK; 29 Department of Biostatistics, Harvard School of Public Health, Boston, USA; 30 Department of Preventive Medicine, Northwestern University, Chicago, USA; 31 Michigan Institute for Clinical and Health Research, University of Michigan, Ann Arbor, USA; 32 Laboratory of Informatics and Systems Engineering for Clinical Research, Istituti Clinici Scientifici Maugeri SpA SB IRCCS, Pavia, Italy; 33 Computational Health Informatics Program, Boston Children’s Hospital, Boston, USA; 34 VA Informatics and Computing Infrastructure, Tennessee Valley Healthcare System Veterans Affairs Medical Center, Nashville, USA; 35 Department of Biostatistics, Epidemiology, and Biostatistics, University of Pennysylvania Perelman School of Medicine, Philadelphia, USA; 36 Department of Biomedical informatics, WiSDM, National University Health Systems Singapore, Singapore, Singapore; 37 Department of Internal Medicine, Division of Medical Informatics, University of Kansas Medical Center, Kansas City, USA; 38 Office of Research and Development, Department of Veterans Affairs, Washington, DC, USA; 39 Data Analytics Center, University of Pennsylvania Health System, Philadelphia, USA; 40 Department of Medicine, National University Hospital, Singapore, Singapore, Singapore; 41 Department of Medical and Surgical Sciences, Infectious and Tropical Disease Unit, University Magna Graecia of Catanzaro, Italy, Catanzaro, Italy; 42 Department of Biomedical Informatics, University of Kentucky, Lexington, USA; 43 Health Catalyst, INC., Cambridge, USA; 44 BIOMERIS (BIOMedical Research Informatics Solutions), Pavia, Italy; 45 Clinical Research Unit of Botucatu Medical School, São Paulo State University, Botucatu, Brazil; 46 Pediatric emergency Department, Hôpital Necker-Enfants Malades, Assistance Public-Hôpitaux de Paris, Paris, Paris, France; 47 National Center for Infectious Diseases, Tan Tock Seng Hospital, Singapore, Singapore, Singapore; 48 BIG-ARC, The University of Texas Health Science Center at Houston, School of Biomedical Informatics, Houston, USA; 49 Department of Pediatrics, Medical University of South Carolina, Charleston, USA; 50 Internal Medicine Department, Botucatu Medical School, São Paulo State University, Botucatu, Brazil; 51 Department of Surgery, St. Luke’s University Health Network, Bethlehem, USA; 52 Department of Medicine, Division of Nephrology, Ente Ospedaliero Cantonale, Lugano, Switzerland; 53 IT Department, Innovation & Data, APHP Greater Paris University Hospital, Paris, France; 54 IT Department, ASST Pavia, Voghera, Italy; 55 Strategy and Transformation Department, APHP Greater Paris University Hospital, Paris, France; 56 Technical University of Munich, Munich, Germany; 57 Scientific Direction, IRCCS Ca’ Granda Ospedale Maggiore Policlinico di Milano, Milan, Italy; 58 North Carolina Translational and Clinical Sciences (NC TraCS) Institute, UNC Chapel Hill, Chapel Hill, USA; 59 Division of Infectious Diseases I, Fondazione I.R.C.C.S. Policlinico San Matteo, Italy, Pavia, Italy; 60 Department of Cardiology, Boston Children’s Hospital, Harvard Medical School, Boston, USA; 61 Department of Biomedical Informatics, HEGP, APHP Greater Paris University Hospital, Paris, France; 62 Department of Anesthesia, St. Luke’s University Health Network, Bethlehem, USA; 63 Université Paris-Saclay, Inria, CEA, Palaiseau, France; 64 INRIA Sophia-Antipolis – ZENITH team, LIRMM, Montpellier, France; 65 Computational Health Informatics Program, Boston Children’s Hospital, Boston, USA; 66 Department of Internal Medicine, University of Kentucky, Lexington, USA; 67 UOC Ricerca, Innovazione e Brand reputation, ASST Papa Giovanni XXIII, Bergamo, Bergamo, Italy; 68 Population Health and Data Science, MAVERIC, VA Boston Healthcare System, Boston, USA; 69 Informatics Institute, University of Alabama at Birmingham, Birmingham, USA; 70 IAM unit, INSERM Bordeaux Population Health ERIAS TEAM, Bordeaux University Hospital/ERIAS - Inserm U1219 BPH, Bordeaux, France; 71 Biomedical Informatics Center, Medical University of South Carolina, Charleston, USA; 72 Clinical Research Informatics, Boston Children’s Hospital, Boston, USA; 73 IT department, Innovation & Data (APHP), UMRS1142 (INSERM), APHP Greater Paris University Hospital, INSERM, Paris, France; 74 Department of Biomedical and Health Informatics, Children’s Hospital of Philadelphia, Philadelphia, USA; 75 Department of Biostatistics, Harvard T.H. Chan School of Public Health, Boston, USA; 76 SED/SIERRA, Inria Centre de Paris, Paris, France; 77 Department of Medicine, Massachusetts General Hospital, Boston, USA; 78 Health Information Technology & Services, University of Michigan, Ann Arbor, USA; 79 Heinrich-Lanz-Center for Digital Health, University Medicine Mannheim, Heidelberg University, Mannheim, Germany; 80 Department of Computational Biology and Bioinformatics, University of Michigan, Ann Arbor, USA; 81 Biostatistics, Perelman School of Medicine at the University of Pennsylvania, Philadelphia, USA; 82 Department of Anesthesiology, Critical Care, and Pain Medicine and Computational Health Informatics Program, Boston Children’s Hospital, Boston, USA; 83 Department of Learning Health Sciences, University of Michigan Medical School, Ann Arbor, MI USA; 84 Institute of Digitalization in Medicine, Faculty of Medicine and Medical Center, University of Freiburg, Germany, Freiburg, Germany; 85 Institute of Medical Biometry and Statistics, Institute of Medical Biometry and Statistics, Medical Center, University of Freiburg, Freiburg, Germany; 86 Department of Biomedical Informatics, HEGP, APHP Greater Paris University Hospital, Paris, France; 87 Division of Biomedical Informatics (Department of Internal Medicine), University of Kentucky, Lexington, USA; 88 Center for Precision Psychiatry, Massachusetts General Hospital, Boston, USA; 89 Department of Critical Care Medicine, Children’s Hospital of PIttsburgh, Pittsburgh, USA; 90 Medical University of South Carolina, Charleston, USA; 91 Department of Pediatrics, Division of Human Genetics, The Children’s Hospital of Philadelphia and the Perelman School of Medicine at the University of Pennsylvania, Philadelphia, USA; 92 Center for Medical Information and Communication Technology, University Hospital Erlangen, Erlangen, Germany; 93 Renaissance Computing Institute/Department of Computer Science, University of North Carolina, Chapel Hill, USA; 94 Clinical Research Unit, Saint Antoine Hospital, APHP Greater Paris University Hospital, Paris, France; 95 Clevy.io, Paris, France; 96 Department of Biostatistics, Harvard T. H. Chan School of Public Health, Boston, USA; 97 Department of Biostatistics, Epidemiology and Informatics, University of Pennsylvania Perelman School of Medicine, Philadelphia, USA; 98 Institute of Medical Biometry and Statistics, Faculty of Medicine and Medical Center, University of Freiburg, Freiburg, Germany, Freiburg, Germany; 99 Michigan Institute for Clinical and Health Research (MICHR) Informatics, University of Michigan, Ann Arbor, MI USA; 100 Harvard Catalyst, Harvard Medical School, Boston, USA; 101 Clinical Research Unit, Paris Saclay, APHP Greater Paris University Hospital, Boulogne-Billancourt, France; 102 Medical Informatics Center, Hospital das Clínicas, Faculty of Medicine of Botucatu, Clinics hospital of the Botucatu Medical School, São Paulo State University, Botucatu, Brazil; 103 Department of Surgery, Beth Israel Deaconess Medical Center, Boston, USA; 104 Department of Anesthesiology and Critical Care, Children’s Hospital of Philadelphia, Philadelphia, USA; 105 ENS, PSL University, Paris, France; 106 Department of Medical and Surgical Sciences, University Magna Graecia of Catanzaro, Italy, Catanzaro, Italy; 107 Internal Medicine Department of Botucatu Medical School, São Paulo State University, Botucatu, Brazil; 108 IAM unit, Bordeaux University Hospital, Bordeaux, France; 109 Department of Biomedical Health Informatics, Children’s Hospital of Philadelphia, Philadelphia, USA; 110 Department of Computational Biomedicine, Cedars-Sinai Medical Center, West Hollywood, USA; 111 Pediatric Infectious Disease Department, Hospital Universitario 12 de Octubre, Madrid, Spain; 112 Department of Biostatistics, Epidemiology, and Informatics (dept), Institute for Biomedical Informatics, University of Pennsylvania Perelman School of Medicine, Berwyn, USA; 113 Department of Infectious Diseases, Great Ormond Street Hospital for Children, UK, London, UK; 114 Department of Neurology, Massachusetts General Hospital, Boston, USA; 115 Harvard Catalyst | The Harvard Clinical and Translational Science Center, Harvard Medical School, Boston, USA; 116 Department of Psychiatry, University of Pennsylvania Perelman School of Medicine, Philadelphia, USA; 117 Computational Health Informatics Program and Department of Pediatrics, Boston Children’s Hospital and Harvard Medical School, Boston, USA; 118 Department of Computational Medicine & Bioinformatics, Internal Medicine, Human Genetics, and School of Public Health, University of Michigan, Ann Arbor, USA; 119 CTSI, WFBMI, Wake Forest School of Medicine, Winston Salem, USA; 120 Department of Internal Medicine, Division of Medical Informatics, University of Kansas Medical Center, Kansas City, USA; 121 Department of Surgery, Beth Israel Deaconess Medical Center, Harvard Medical School, Boston, USA; 122 NC TraCS Institute, UNC Chapel Hill, Chapel Hill, USA; 123 Department of Medical Informatics, University of Erlangen-Nürnberg, Erlangen, Germany; 124 Clinical Research Unit São Paulo State University, Clinical Research Unit São Paulo State University, Botucatu, Brazil; 125 Department of Veterans Affairs Department of Veterans Affairs, Office of Research and Development, Washington, DC, USA; 126 Division of Infectious Diseases, Department of Medicine II, Medical Center – University of Freiburg, Faculty of Medicine, Freiburg, Germany; 127 Biomedical Data Science Lab, ITACA Institute, Universitat Politècnica de València, Valencia, Spain; 128 Department of Pediatrics (Critical Care), Northwestern University Feinberg School of Medicine, Chicago, USA; 129 Nurse Departament of FMB - Medicine School of Botucatu, Clinical Research Unit of Botucatu Medical School, São Paulo State University, Botucatu, Brazil; 130 ASST Pavia, Lombardia Region Health System, Management Engineer, Direction, Pavia, Italy; 131 Department of Anesthesiology, University Hospital Erlangen, FAU Erlangen-Nürnberg, Germany, Erlangen, Germany; 132 Digital Research, Informatics and Virtual Environments (DRIVE), Great Ormond Street Hospital for Children NIHR BRC, UK, London, UK; 133 Hôpital Saint Louis, Department of Biostatistics and Bioinformatics, APHP Greater Paris University Hospital, Paris, France; 134 MICHR Informatics, University of Michigan, Ann Arbor, USA; 135 CHIP, Boston Children’s Hospital, Boston, USA; 136 Department of Neurology, University of Pittsburgh Medical Center, Pittsburgh, USA; 137 Critical Care Medicine, Department of Medicine, St. Luke’s University Health Network, Bethlehem, PA USA; 138 Department of Pediatrics-Section of Nephrology, Brenner Children’s, Wake Forest University School of Medicine, Winston Salem, USA; 139 Department of Biomedical Health Informatics and the Department of Pediatrics, The Children’s Hospital of Philadelphia and the University of Pennsylvania Perelman Medical School, Philadelphia, USA; 140 Yong Loo Lin School of Medicine, National University of Singapore, Singapore, Singapore; 141 Department of Information Management, National Central University, Taoyuan, Taiwan; 142 Clinical & Translational Science Institute, Medical College of Wisconsin, Milwaukee, USA; 143 Université Paris-Saclay, Inria, CEA, Montréal Neurological Institute, McGill University, Palaiseau, France; 144 SequeL, Inria Lille, Villeneuve-d’Ascq, France; 145 Respiratory Department, ICS S. Maugeri IRCCS Pavia Italy, Lumezzane (Bs), Italy; 146 Department of Health Management and Informatics, University of Missouri, MO Columbia, USA; 147 Department of Neurology, University of Pittsburgh, Pittsburgh, USA; 148 Department of Veterans Affairs, 1100 First Street, NW, Washington, DC, 20420 USA; 149 University of Pittsburgh, Pittsburgh, USA; 150 Department of Anesthesiology and Critical Care Medicine, Children’s Hospital of Philadelphia and University of Pennsylvania, Philadelphia, USA; 151 Department of Oncology, ASST Papa Giovanni XXIII, Bergamo, Bergamo, Italy

**Keywords:** Viral infection, Data mining

## Abstract

Given the growing number of prediction algorithms developed to predict COVID-19 mortality, we evaluated the transportability of a mortality prediction algorithm using a multi-national network of healthcare systems. We predicted COVID-19 mortality using baseline commonly measured laboratory values and standard demographic and clinical covariates across healthcare systems, countries, and continents. Specifically, we trained a Cox regression model with nine measured laboratory test values, standard demographics at admission, and comorbidity burden pre-admission. These models were compared at site, country, and continent level. Of the 39,969 hospitalized patients with COVID-19 (68.6% male), 5717 (14.3%) died. In the Cox model, age, albumin, AST, creatine, CRP, and white blood cell count are most predictive of mortality. The baseline covariates are more predictive of mortality during the early days of COVID-19 hospitalization. Models trained at healthcare systems with larger cohort size largely retain good transportability performance when porting to different sites. The combination of routine laboratory test values at admission along with basic demographic features can predict mortality in patients hospitalized with COVID-19. Importantly, this potentially deployable model differs from prior work by demonstrating not only consistent performance but also reliable transportability across healthcare systems in the US and Europe, highlighting the generalizability of this model and the overall approach.

## Introduction

Severe acute respiratory syndrome coronavirus 2 (SARS-CoV-2) has caused millions of cases of coronavirus disease 2019 (COVID-19) in nearly every country. While most patients with COVID-19 have a mild form of viral pneumonia, an appreciable subgroup develops rapid onset of severe disease. Several large national studies have demonstrated that a variable and potentially significant proportion (ranging from 5% to 70%)^1–3^ of hospitalized patients with COVID-19 develop cardiorespiratory failure, require mechanical ventilation and hemodynamic support, and may ultimately die. The early identification of patients at high risk for death can improve triage and resource allocation, particularly when numbers of COVID-19 cases overwhelm health systems^4^.

Numerous studies have reported models using clinical data, including laboratory values, to predict patients at high risk of death for COVID-19^2^. However, most models have not been tested across hospital systems and countries to determine generalizability. Few studies have included patients from multi-national cohorts. The international nature of this disease begs the question of whether models derived using data from one site or one country can be used in another. Is transportability possible if the experience of one site or country could help another make better decisions?

We formed the 4CE Consortium^5^ as an international research collaborative of nearly 300 hospitals from four countries in order to collect standardized patient-level electronic health record (EHR) data to examine the epidemiology, pathophysiology, management, and healthcare system dynamics of COVID-19. Using the 4CE data, we examined the relationship between pre-selected laboratory values^6^ and mortality across institutions and countries. We compared prediction models using single laboratory values at admission to a prediction model containing multiple laboratory values. Across all models, we evaluated geographical differences (national and continental) among the outcome prediction models to better understand if models trained on data from one country and institution can be used elsewhere.

## Results

### Characteristics of the study population

**Fig. 1:**
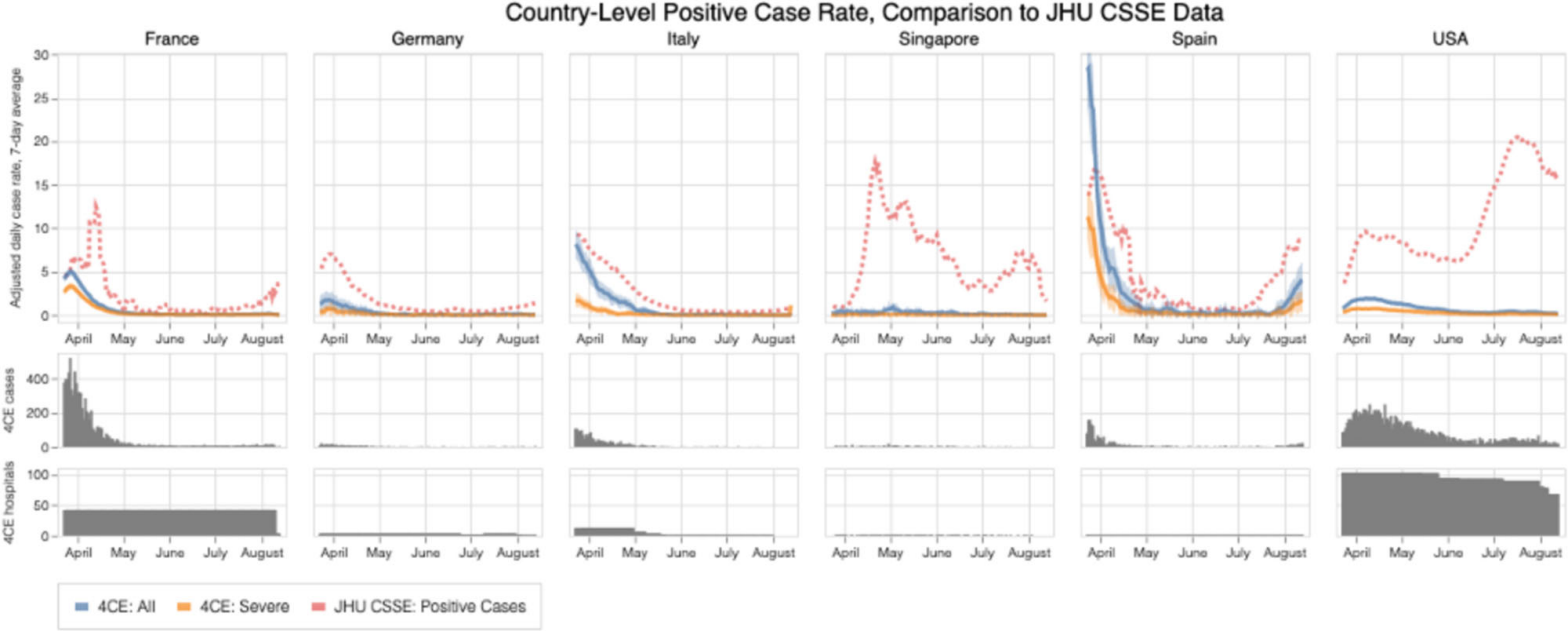
Comparison of National Hospitalization Rates by Data Source. Adjusted 7-day average new hospitalization rate and rate of ever-severe disease per 100,000 people by country based on 4CE contributors along with 95% confidence intervals compared with 7-day average new case rates collected by Johns Hopkins Center for Systems Science and Engineering (JHU CSSE).

In this study population of 39,969 patients, the incidence of hospitalization for COVID-19 largely tracked with population dynamics of COVID-19 cases^7^ across different countries during the initial pandemic period (Fig. 1). Both the COVID-19 case rate and the COVID-19 hospitalization rate dropped significantly from the first peak in April 2020. While hospitalization rates remained relatively low for all countries, case rates increased in France, Germany, Spain and United States after June 2020.

Consistent with prior studies^4,8^, the study population of patients hospitalized with COVID-19 showed a higher prevalence of men and older populations. See Supplementary Fig. 1 for demographic characteristics and percentages among age group, race/ethnicity, and sex. International comparisons were consistent and showed across three countries that most patients (79.6%) were 50 years of age or older and male (68.6%).

### International comparisons of individual laboratory tests at admission for mortality risk prediction

The prediction performances of individual laboratory test across all sites, at country level and continent level were summarized using random-effects meta-analysis. On average, albumin, creatinine, neutrophil count, CRP and white blood cell were stronger predictors of mortality than the other labs (Supplementary Fig. 2). The predictiveness of the laboratory tests for mortality within the next few days after admission tends to be slightly higher than for 1 or 2-week mortality although the decrease in predictiveness over time was moderate. The predictiveness of the labs varies substantially across sites. Albumin has low predictiveness in European sites but higher in the US, CRP appears to be slightly more predictive in Europe than in US, while other labs performed similarly in the US and in Europe on average.

### International comparisons of mortality risk prediction model

**Fig. 2:**
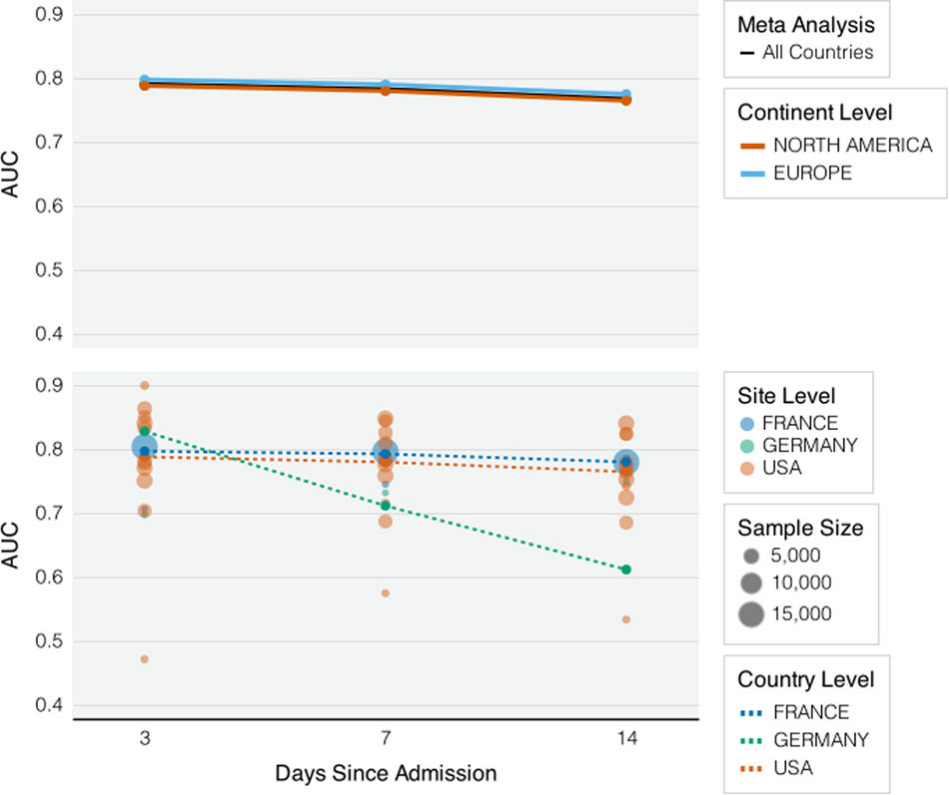
Risk Model Performance Across Countries and Continents. AUCs of cox regression models with nine common laboratory tests (missing rate <30%) in predicting death adjusting for demographic variables and Charlson comorbidity index.

The estimated log hazard ratios for demographic, nine laboratory tests and Charlson comorbidity index from a comprehensive Cox model are largely consistent across different healthcare systems with respect to their directions and magnitudes (Supplementary Fig. 3). The estimated log hazard ratios across all sites and at country level were summarized using random-effects meta-analysis. The risk models indicate that age, albumin, AST, creatine, CRP, and white blood cell are most predictive of mortality. For example, the risk model predicts a protective effect against mortality from those who are <50 years old, report higher albumin values and lymphocyte count values, and report lower AST, creatinine and CRP values. The average AUC of the full risk model is about 0.80, 0.79 and 0.77 for predicting both 3-day, 1-week, or 2-week mortality (Fig. 2). While the performance of the locally trained site-level models varies across healthcare systems, the average performance of the full model is similar in the US versus Europe.

### Portability of mortality algorithms across sites, countries, and continents

**Fig. 3:**
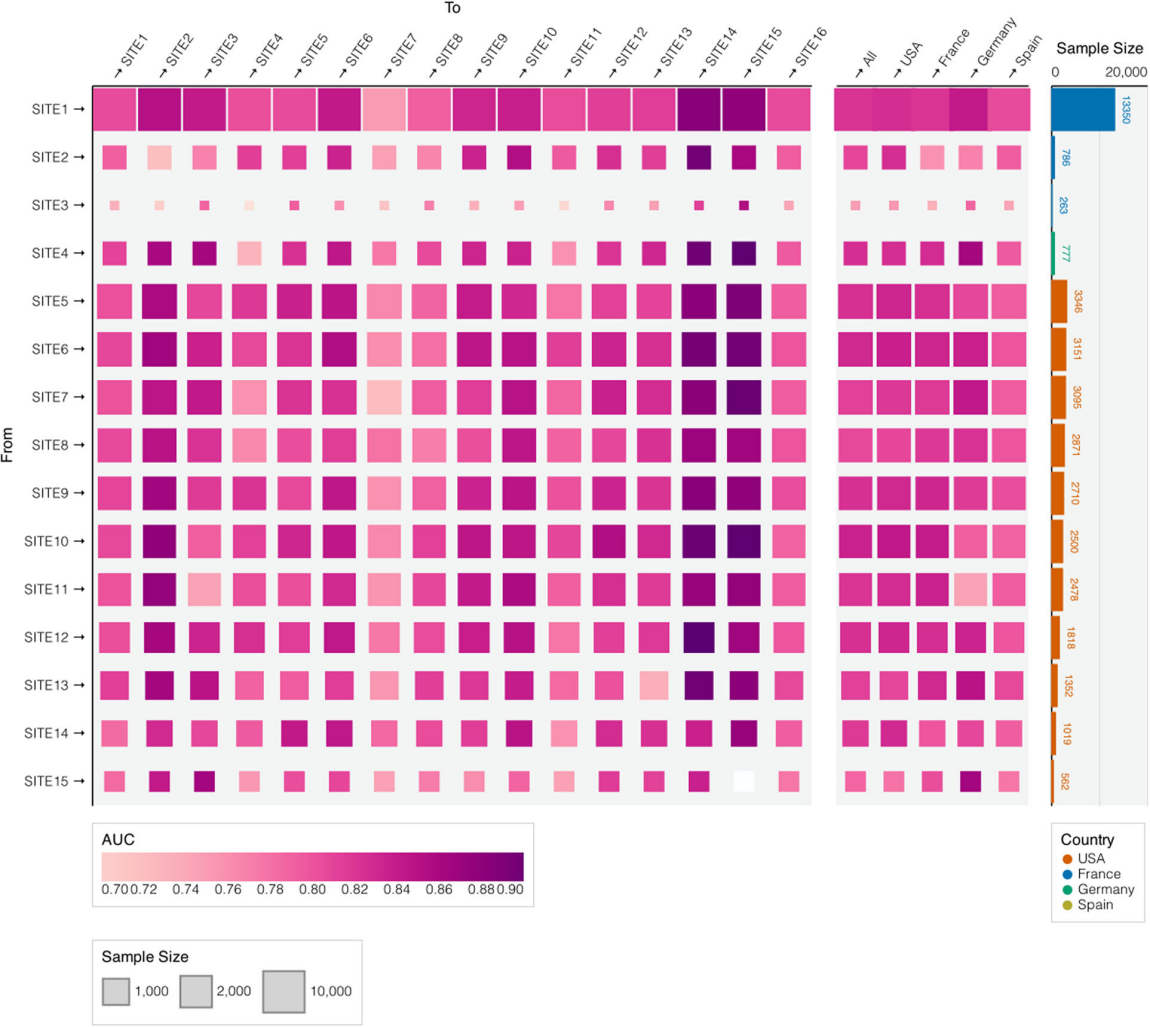
Transportability of the Mortality Prediction Model Across Sites and Countries. Heatmap of transportability of the Cox regression model across different sites and countries. Each part of the figure represents performance when the model is trained at one site and evaluated at another.

The AUCs of the locally trained mortality risk models for 1-week mortality when porting to external sites were summarized in Fig. 3 (refer to Supplementary Table 4 for numerical results). The averaged AUCs across all sites and at country level were summarized using random-effects meta-analysis. The algorithms trained from sites with large cohort size tend to have better performance both locally and when transported to other sites. For example, the AUCs of the model trained at SITE1 (France) are always close to or higher than the those of the local trained model. We additionally compared the portability performance across continents. In general, when porting to North America sites, the algorithms trained at both continents perform equally well. For example, when porting to SITE5 (US), the maximum AUC was 0.842 and 0.847 for algorithms trained at North America sites and at European sites, respectively, which are very close to the maximum AUC of the local SITE5 algorithm. On the other hand, when porting to Europe sites, the algorithms trained at North America sites perform slightly better than those trained at Europe sites, due to the relatively smaller sample size of the Europe sites. For example, when porting to SITE1 (France), the maximum AUC was 0.813 and 0.791 for algorithms trained at North America sites and at European sites, respectively.

## Discussion

In this large-scale multi-national study, we reported a mortality prediction model for patients hospitalized with COVID-19 that retained accuracy across healthcare systems and countries. Building on the growing literature of COVID-19 mortality prediction, our study is unique in leveraging international cohorts to validate the generalizability of the prediction model, which has the following specific features. First, a predictive model containing nine commonly measured laboratory test values performed better than the model containing 17 laboratory test values: CRP, creatinine, white blood cell count, lymphocyte count, AST, ALT, total bilirubin, neutrophil count, and albumin. From a list of 17 laboratory tests associated with worse outcomes in patients with COVID-19 based on prior reports^6^, we selected the subset of nine tests based on their low rate of missing data in our data set. Second, we identified albumin, CRP, creatinine, neutrophil count, and white blood cell count as better individual predictors than other individual laboratory tests. Third, a comprehensive model containing the nine commonly measured laboratory tests as well as baseline demographic features and comorbidity burden indicates that age, albumin, AST, CRP, creatine, and white blood cell count are most predictive of mortality. Interestingly, the baseline covariates are more predictive of mortality in the early days after admission for COVID-19, likely because other features gain importance as hospital course prolongs. Finally, when comparing prediction models between North American and European sites, the final model showed crucial consistency across international sites, highlighting its potential generalizable application.

The study has several strengths. Chief among them is the international consortium with a federated data sharing approach that facilitated the pooling of laboratory values across 283 hospitals with diverse healthcare practices and populations, enabling the examination of model transportability. Second, while the accuracy (AUC) of individual laboratory test in predicting mortality after hospital admission for COVID-19 varies substantially cross countries, the accuracy of the mortality risk prediction model is remarkably consistent between US and Europe. Further, the estimated log hazard ratios from the best-performing Cox model are largely consistent across different healthcare systems with respect to their directions and magnitudes. Third, the mortality prediction model using commonly measured laboratory tests and baseline demographic and comorbidity burden trained at healthcare systems performs well both locally and externally when transported to other sites. Interestingly, the transportability does not appear to depend on the continent or country. Taken together, the key innovation of our study that differs from prior studies is the transportability and the potential generalizability of the COVID-19 mortality prediction model that seems independent of the specific healthcare system.

The study also has several limitations that we took measures to mitigate. First, EHR data have variable degree of intrinsic noise, missing data, and available documentation due to differences in clinical practice that contribute to differences among healthcare systems. Indeed, we found healthcare system-level (within-healthcare system and between-healthcare system) differences were greater than country-level differences. By leveraging our federated system of common EHR data elements and capturing healthcare system-level heterogeneity, the 4CE consortium is uniquely positioned to identify international differences in patient characteristics and outcomes as well as to test model transportability. To mitigate the quality issue of EHR data, we performed extensive and iterative quality controls at *each* participating healthcare system with local collaborators and centrally to address potential imprecision due to healthcare system-specific variations in data extraction and incompleteness of datasets (e.g., incomplete mapping of local EHR codes to desired data elements). These critical quality control steps, which are often underappreciated in multi-center EHR data research, further differentiate the 4CE research efforts from other COVID-19 research efforts. Second, we observed a significant level of heterogeneity in the predictiveness of individual laboratory tests and the locally trained mortality risk models across the participating healthcare systems. The heterogeneity could result from differences in patient population, clinical practice and EHR system. To address this concern, we performed random-effects meta-analyses to account for the heterogeneity across sites. Importantly, the best-performing model showed evidence of good transportability despite of the heterogeneity.

As the pandemic persists and new SARS-CoV-2 variants emerge, two clinically relevant questions remain unanswered: (1) does the mortality prediction model continue to perform well across healthcare systems and countries? (2) can the prediction model predict long-term mortality after COVID-19 hospitalization? To address these questions, we are planning future analyses using patient-level data at each participating healthcare system to assess the temporal trends of the model performance throughout the pandemic waves and at individual patient-level over longer period. We will revise and adapt to temporal changes in clinical scenarios. In this study, we observed that AUCs are generally consistent across genders. Since age is a significant risk factor for mortality, conditioning on the age group, the model performance for distinguishing high-risk vs. low-risk patients within the age group is expected to be lower than the overall accuracy. Further developing age-specific risk prediction models warrants further research. Beyond mortality prediction, the 4CE consortium has established a platform of harmonized data capture through its federated system with iterative and methodical expansion of data elements to enable the clinical investigation of a wide range of domains pertaining to COVID-19 such as coagulopathy and thrombotic events, acute renal failure, pediatric manifestation, neurological complications as well as the post-acute sequelae syndrome (i.e., long-hauler). We will apply the approach from this study to assess other prediction model transportability within our international network of participating healthcare systems.

We make several noteworthy observations of clinical relevance. First, the laboratory tests predictive of mortality in patients hospitalized for COVID-19 represent the combination of acute inflammatory response (as indicated by CRP, white blood cell, lymphocyte, and neutrophil count) and underlying physiological function as well as the acute response of critical organ systems (general nutritional status as indicated by albumin, renal function as indicated by creatinine, and hepatic function as indicated by AST, ALT, and bilirubin). These routinely collected laboratory indicators of systemic response to the SARS-CoV-2 viral infection in conjunction with easily ascertainable baseline demographic and comorbidity burden formulate a clinically deployable prediction tool of mortality risk following hospital admission for COVID-19. Second, the relatively modest accuracy of individual laboratory values in predicting mortality is likely due to its large variation within each participating healthcare system. This combination of commonly measured clinical laboratory tests dramatically improved the prediction performance over individual laboratory tests, and performed better than a larger panel of clinical laboratory tests. A key clinical insight is that clinical laboratory tests beyond the commonly measured routine tests may not inform mortality, which is the most important clinical outcome. Third, the performance of the final model was relatively stable over the hospital course and did not improve beyond the initial hospital days. This finding suggests that additional factors contribute to mortality as the hospital course for COVID-19 patients prolongs. Of particular clinical relevance, it supports the utility of commonly measured routine clinical laboratory test values (and other routine clinical and demographic features) at admission to identify patients at high risk for mortality who would warrant early and aggressive intervention as well as close monitoring, particularly in the setting of limited healthcare resources.

## Methods

### Cohort identification

We included all patients hospitalized at participating 4CE sites with an admission date from 7 days before to 14 days after the date of their first reverse transcription polymerase chain reaction (PCR)-confirmed SARS-CoV-2 positive test result. The first admission date within this 21-day time window was considered the index admission date. Throughout this work, “days since admission” refers to this index date.

### Participating sites

Data were available from 39,969 patients from 284 hospitals (affiliated with 16 sites) across four countries: France, Germany, Spain, and the United States. See Supplementary Table 2 for details about participating sites. Several sites collected data from multiple hospitals. In the United States, 170 medical centers of the US Department of Veterans Affairs were grouped into five regional divisions called Veterans Integrated Service Networks.

### Patient and public involvement

Patients and the public were not involved in the design, conduct, or reporting, or dissemination plans of the research.

### Outcome

We consider death as the main COVID-19 outcome. Death was identified via standard coding and discharge data aggregation from each site. Each partner institution used local criteria to identify in-hospital mortality.

### Local data collection

#### Patient-level data

**Fig. 4:**
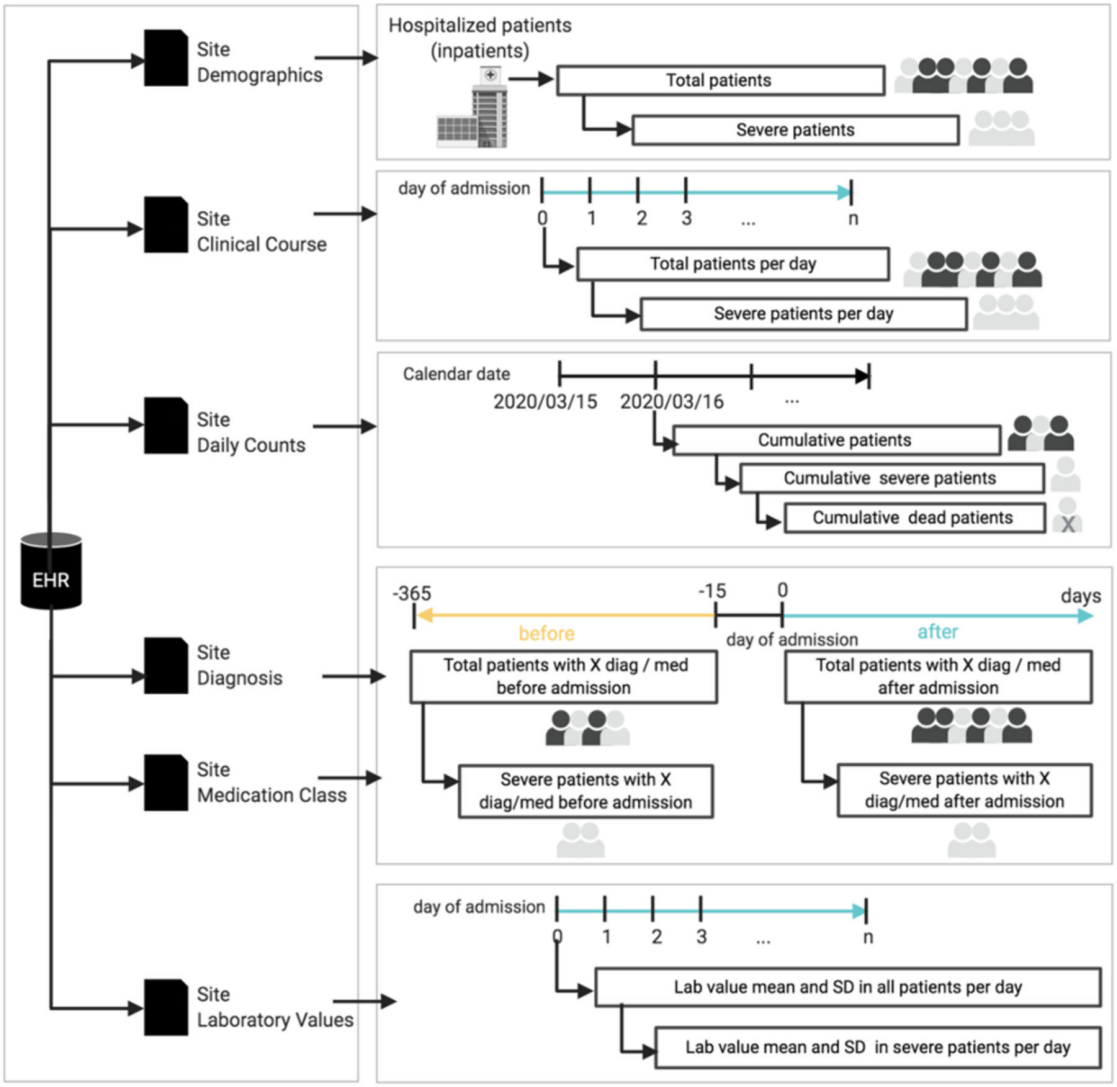
Schematic of the federated EHR-based study involving healthcare systems from three countries. Each site generated three data tables (comma-separated files) containing patient level data: 1) local patient clinical course indicates which days the patient was in the hospital and when the patient died; 2) local patient observation includes first three-character ICD9/10 diagnosis code and laboratory tests, where laboratory test has a numerical value; 3) local patient summary contains demographic variables including age, sex and race. Sites then conduct analysis using these individual level data within their firewall (see Methods).

Sixteen sites representing 284 Hospitals assembled patient-level data for detailed analyses, including twelve US sites, and four international sites. Individual healthcare systems then ran separate analyses using the patient-level data within their local firewall and only reported the final analytic results to the central institution for meta-analysis. A schematic of our workflow is presented in Fig. 4, and further details of collected data are reported in Supplementary Table 3.

#### Software platform

Most sites used the open source i2b2 (Informatics for Integrating Biology and the Bedside) software platform to obtain the data. More than 200 organizations worldwide use i2b2 for purposes that include identifying participants for clinical trials, drug safety monitoring, and clinical and epidemiological research. Those 4CE sites with i2b2 used database scripts to directly query their i2b2 repository, calculate the counts and statistics, and export the data files. The 4CE sites without i2b2 used the Observational Medical Outcomes Partnership (OMOP) Common Data Model or their own clinical data warehouse solutions (e.g., Epic Caboodle) and querying tools to create the required files.

### Selection of laboratory tests

We focused on nine laboratory tests that are commonly measured (missing rate <30% at most sites) and associated with mortality in patients with COVID-19 based on prior reports^6^, We provided each site with a single standard Logical Objects, Identifiers, Names and Codes (LOINC) identifier for each test, but sites often needed to map tests to additional LOINC or custom codes within their EHR. We addressed barriers that arose during initial efforts to extract these laboratory values by stratifying region-specific laboratory test types to reduce extraction errors and enable standardization.

### Quality control

We conducted site-specific quality control. Each site ran an R script for the following additional quality control checks: consistency of the total counts of total cases across all datasets within each site, consistency between the 3-digit diagnosis codes and the ICD dictionary, and consistency of the range of laboratory data from each site with the normal range observed from all sites. Sites checked and fixed the data if their laboratory values were consistently lower or higher than the other sites or otherwise implausible.

### Statistical analysis

We estimated the country-level daily incidence of new patients hospitalized with COVID-19 during the study period from March 1, 2020 to September 30, 2020. Specifically, for each country, we summed the daily incidence of new patients hospitalized with COVID-19 at each site within that country per 100,000 people of the country and multiplied this by an adjustment factor, defined as the ratio between the country’s overall inpatient discharge rate and the overall inpatient discharge rate of all 4CE sites in that country irrespective of COVID-19 status. We then reported the adjusted 7-day average incidence of new COVID-19 hospitalizations per 100,000 of the country population.

We divided our analysis into two parts: (1) prediction of mortality using individual laboratory values and a comprehensive algorithm derived from multiple laboratory values, comorbid conditions, and demographics available at each site and (2) comparison of these models across sites, countries, and continents.

We evaluated the ability of a biomarker and demographics-based algorithm to predict mortality using admission data. We removed patients who died at admission. We developed mortality risk prediction models using a set of nine common laboratory tests with missing rates <30% at most sites, adjusting for demographic variables and the Charlson comorbidity index. We derived the risk models by fitting penalized Cox proportional hazards model. We evaluated the accuracy of the risk models for predicting mortality by t-days since admission based on the time-specific AUC^9^. We used the 10-fold cross-validation to estimate the AUC when evaluating the model performance within each local site. The mortality risk prediction model was not trained at Spain because the data were not available at the time when we collected the model training results. To assess the transportability of the mortality risk prediction models across different sites, we validated the algorithm trained at local individual healthcare centers using independent dataset from remaining external sites including the healthcare center from Spain. We used random effects meta-analysis on the prediction performance measures across sites to summarize country level, continent level, and overall average performances.

IRB Approval was obtained at Assistance Publique—Hôpitaux de Paris, Beth Israel Deaconess Medical Center, Bordeaux University Hospital, Hospital Universitario 12 de Octubre, Massachusetts General Brigham, Northwestern University, Medical Center, University of Freiburg, University of Pittsburgh, VA North Atlantic, VA Southwest, VA Midwest, VA Continental, and VA Pacific. An exempt determination was made by the IRB at University of California Los Angeles, University of Michigan, and University of Pennsylvania.

### Reporting summary

Further information on research design is available in the Nature Research Reporting Summary linked to this article.

## Supplementary information

Supplemental Material

Reporting Summary

## Data Availability

Only aggregate data was shared by sites for this study. All aggregate data in a de-identified fashion can be found and downloaded at www.covidclinical.net.

## Code availability

The SQL and R scripts used in this work can be found and downloaded at https://github.com/covidclinical.
