# Supplementary material for: International comparisons of laboratory values from the 4CE collaborative to predict COVID-19 mortality
Source: NPJ Digit Med. 2022 Jun 13;5:74. doi: 10.1038/s41746-022-00601-0 (PMC9192605; doi:10.1038/s41746-022-00601-0)
Supplement: Supplementary file 1 — Supplemental Material [file 41746_2022_601_MOESM1_ESM.docx]

**Supplementary Materials for “International Comparisons of Laboratory Values from the 4CE Collaborative to Predict COVID-19 Mortality**

**”**

1. **Supplementary Figures**
   1. **Supplementary Figure 1. Distribution of demographic groups stratified by country.**
   2. **Supplementary Figure 2: AUCs of individual laboratory test result at admission in predicting death at site level, country level and continent level.**
   3. **Supplementary Figure 3: Coefficients of the Cox model with nine common laboratory tests.**
   4. **Supplementary Figure 4: Sensitivities and PPV of the Cox model with nine common labs in predicting death at site level, country level and continent level. The cutoff was chosen at specificity=0.9.**
   5. **Supplementary Figure 5**. **AUCs of cox regression models with nine common laboratory tests (missing rate <30%) in predicting death adjusting for demographic variables and Charlson comorbidity index stratified by gender and age.**
   6. **Supplementary Figure 6**. AUCs of cox regression models when transporting to other sites.
2. **Supplementary Tables**
   1. **Supplementary Table 1: Membership of 4CE.**
   2. **Supplementary Table 2: Participating sites.**
   3. **Supplementary Table 3: Four comma-separated values (CSV) files and their descriptions.**
   4. **Supplementary Table 4**: AUCs of cox regression model across different sites, countries, and continents.
3. **Supplementary Figures**
4. Age


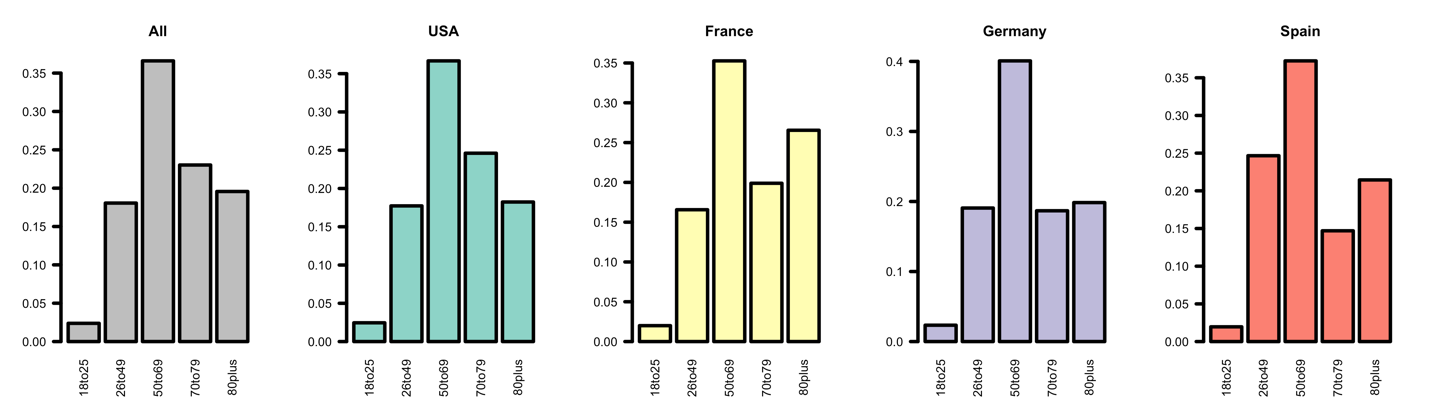


1. Gender


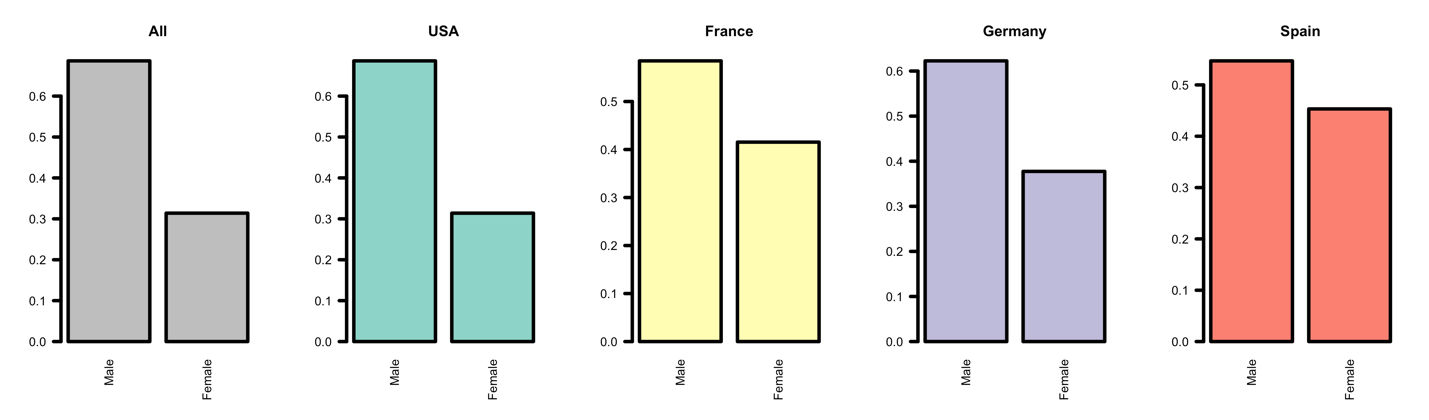


1. Race

**
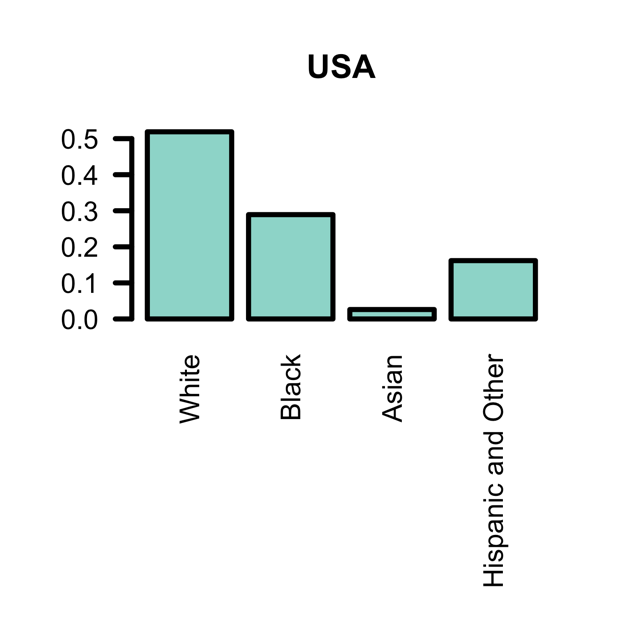
**

**Supplementary Figure 1.** Distribution of demographic groups stratified by country.


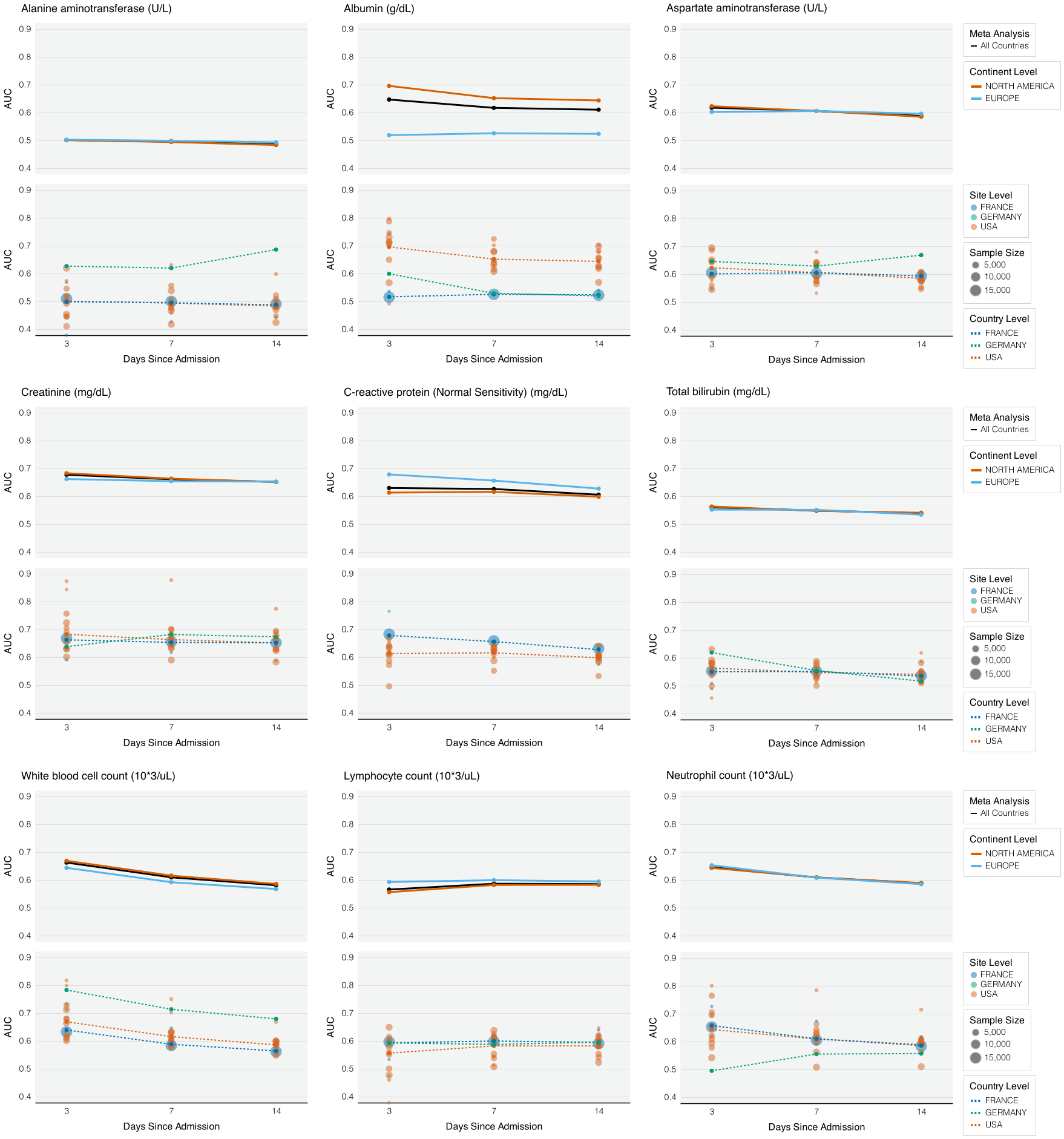


**Supplementary Figure 2**. AUCs of individual laboratory test result at admission in predicting death at site level, country level and continent level.


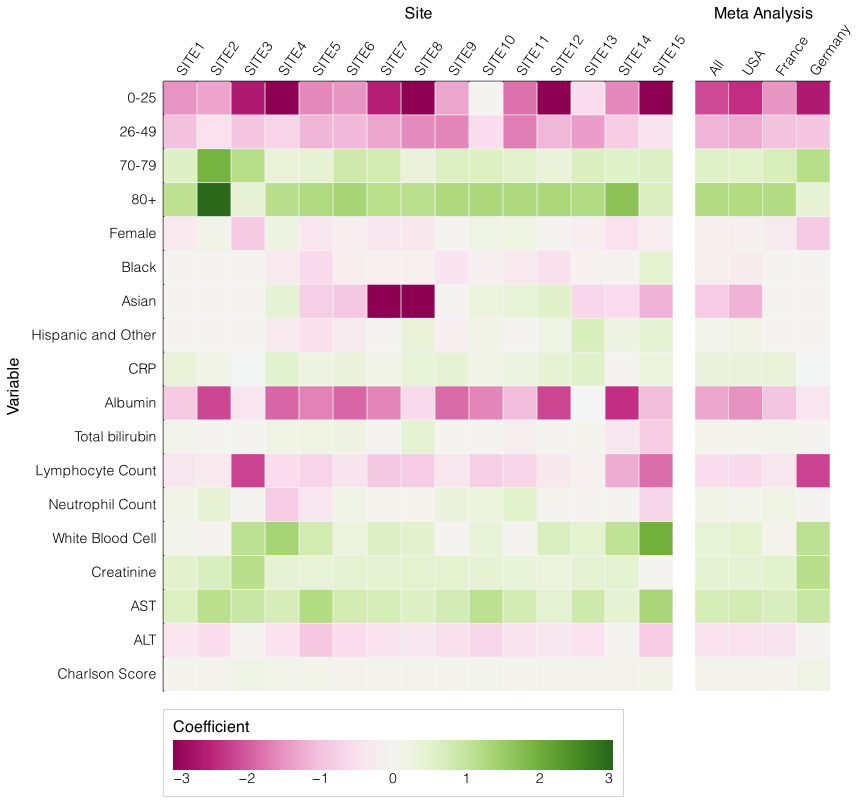


**Supplementary Figure 3**. Coefficients of the Cox model with nine common laboratory tests, standard demographic variables and Charlson comorbidity index.

(a) Sensitivities


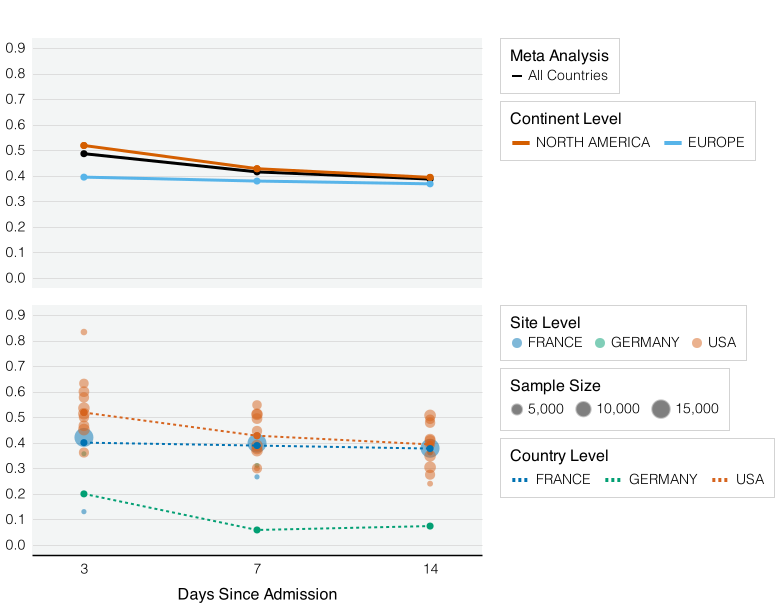


(b) PPV


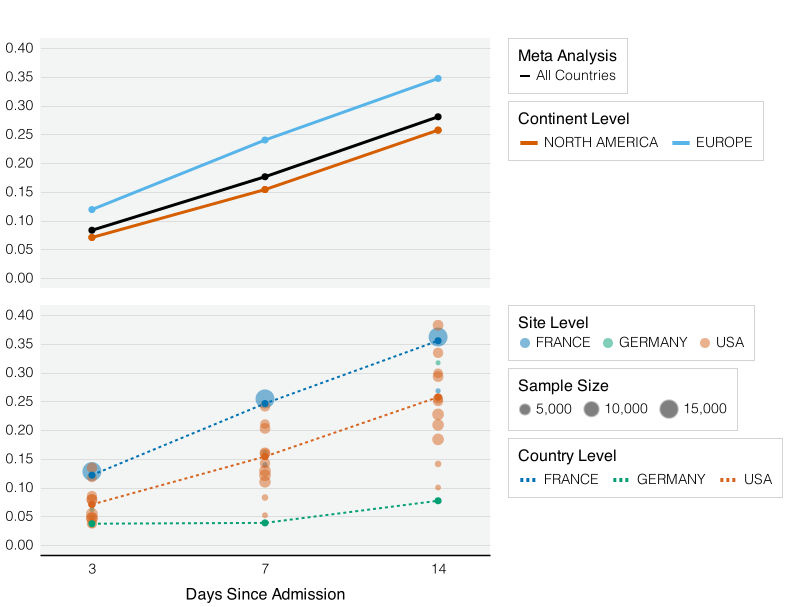


**Supplementary Figure 4**. Sensitivities and PPV of the Cox model in predicting death at site level, country level and continent level. The cutoff was chosen at specificity=0.1.

1. Stratified by gender

1. Stratified by age

**Supplementary Figure 5**. AUCs of cox regression models with nine common laboratory tests (missing rate <30%) in predicting death adjusting for demographic variables and Charlson comorbidity index stratified by gender and age.

**
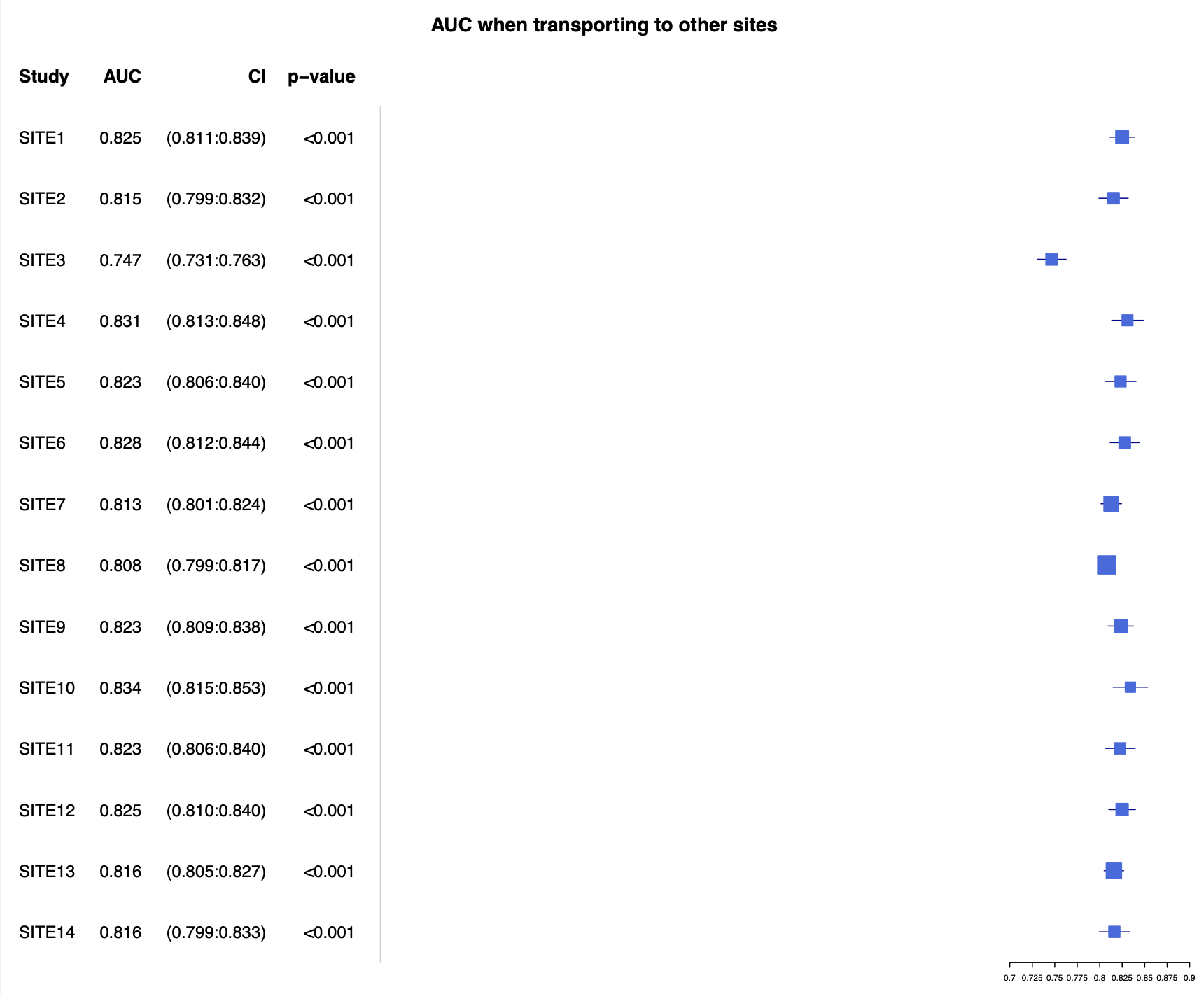
**

**Supplementary Figure 6**. AUCs of cox regression models when transporting to other sites.

**IV. Supplementary Tables**

**Supplementary Table 1**: Membership of 4CE.

| First Name | **Middle Initial** | **Last Name** | **Degree(s)** | **Email Address** | **Primary Affiliation - Institution** | **Country** |
| --- | --- | --- | --- | --- | --- | --- |
| James | R | Aaron | MHA | james.aaron@uky.edu | University of Kentucky | United States |
| Giuseppe |  | Agapito | PhD | agapito@unicz.it | University Magna Graecia of Catanzaro, Italy | Italy |
| Adem |  | Albayrak |  | adem.albayrak@healthcatalyst.com | Health Catalyst, INC. | United States |
| Mario |  | Alessiani | MD, FACS | [m.alessiani@unipv.it](mailto:m.alessiani@unipv.it) | ASST Pavia, Lombardia Region Health System | Italy |
| Danilo | F | Amendola | MD | daniloupeclin@gmail.com | Clinical Research Unit of Botucatu Medical School, São Paulo State University, Botucatu, Brazil | Brazil |
| Li | L.L.J | Anthony |  | anthony.li@mohh.com.sg | National Center for Infectious Diseases, Tan Tock Seng Hospital, Singapore | Singapore |
| Bruce | J | Aronow | PhD | bruce.aronow@cchmc.org | Cincinnati Children's Hospital Medical Center, University of Cincinnati | United States |
| Andrew |  | Atz | MD | atzam@musc.edu | Medical University of South Carolina | United States |
| Paul |  | Avillach | MD, PhD | paul_avillach@hms.harvard.edu | Harvard Medical School | United States |
| James |  | Balshi |  | James.Balshi@sluhn.org | St. Luke's University Health Network, Bethlehm PA | United States |
| Brett | K | Beaulieu-Jones | PhD | brett_beaulieu-jones@hms.harvard.edu | Harvard Medical School | United States |
| Douglas | S | Bell |  | dbell@mednet.ucla.edu | Department of Medicine, David Geffen School of Medicine at UCLA | United States |
| Antonio |  | Bellasi |  | abellasi@asst-pg23.it | UOC Ricerca, Innovazione e Brand reputation, ASST Papa Giovanni XXIII, Bergamo | Italy |
| Riccardo |  | Bellazzi | MS, PhD | riccardo.bellazzi@unipv.it | University of Pavia, Italy | Italy |
| Vincent |  | Benoit | PhD | vincent.benoit@aphp.fr | APHP Greater Paris University Hospital | France |
| Michele |  | Beraghi |  | michele_beraghi@asst-pavia.it | ASST Pavia | Italy |
| José Luis |  | Bernal Sobrino | MS | joseluis.bernal@salud.madrid.org | Hospital Universitario 12 de Octubre, Madrid, Spain | Spain |
| Mélodie |  | Bernaux |  | melodie.bernaux@aphp.fr | APHP Greater Paris University Hospital | France |
| Romain |  | Bey |  | romain.bey@aphp.fr | APHP Greater Paris University Hospital | France |
| Alvar |  | Blanco Martínez | MS | alvar.blanco@salud.madrid.org | Hospital Universitario 12 de Octubre, Madrid, Spain | Spain |
| Martin |  | Boeker |  | martin.boeker@imbi.uni-freiburg.de | Faculty of Medicine and Medical Center, University of Freiburg, Freiburg, Germany | Germany |
| Clara-Lea |  | Bonzel | MSc | clbonzel@hsph.harvard.edu | Harvard Medical School | United States |
| John |  | Booth | MSc | john.booth@gosh.nhs.uk | Great Ormond Street Hospital for Children, UK | United Kingdom |
| Silvano |  | Bosari |  | silvano.bosari@unimi.it | Scientific Direction, IRCCS Ca' Granda Ospedale Maggiore Policlinico di Milano | Italy |
| Florence | T | Bourgeois |  | florence.bourgeois@childrens.harvard.edu | Harvard Medical School | United States |
| Robert | L | Bradford |  | rbrad@med.unc.edu | North Carolina Translational and Clinical Sciences (NC TraCS) Institute, UNC Chapel Hill | United States |
| Gabriel | A | Brat | MD | gbrat@bidmc.harvard.edu | Harvard Medical School | United States |
| Stéphane |  | Bréant |  | stephane.breant@aphp.fr | APHP Greater Paris University Hospital | France |
| Mauro |  | Bucalo |  | mauro.bucalo@biomeris.it | BIOMERIS (BIOMedical Research Informatics Solutions) | Italy |
| Anita |  | Burgun |  | anita.burgun@aphp.fr | APHP Greater Paris University Hospital | France |
| Tianxi |  | Cai | PhD | tcai@hsph.harvard.edu | Harvard Medical School | United States |
| Mario |  | Cannataro | M.Sc. | cannataro@unicz.it | University Magna Graecia of Catanzaro, Italy | Italy |
| Aize |  | Cao |  | aize.cao@va.gov | Tennessee Valley Healthcare System Veterans Affairs Medical Center | United States |
| Aldo |  | Carmona |  | aldo.carmona@sluhn.org | Department of Anesthesia, St. Luke's University Health Network, Bethlehem, PA | United States |
| Charlotte |  | Caucheteux |  |  | Université Paris-Saclay, Inria, CEA | France |
| Julien |  | Champ |  | julien.champ@inria.fr | INRIA Sophia-Antipolis – ZENITH team, LIRMM, Montpellier, France | France |
| Jin |  | Chen | PhD | chen.jin@uky.edu | University of Kentucky | United States |
| Luca |  | Chiovato |  | luca.chiovato@icsmaugeri.it | Unit of Internal Medicine and Endocrinology, Istituti Clinici Scientifici Maugeri SpA SB IRCCS, Pavia, Italy and Department of Internal Medicine and Therapeutics, University of Pavia, Pavia, Italy. | Italy |
| James | J | Cimino |  | ciminoj@uab.edu | UAB Informatics Institute | United States |
| Tiago | K | Colicchio |  | tcolicchio@uabmc.edu | UAB Informatics Institute | United States |
| Sylvie |  | Cormont |  | sylvie.cormont@aphp.fr | APHP Greater Paris University Hospital | France |
| Sébastien |  | Cossin |  | sebastien.cossin@chu-bordeaux.fr | Bordeaux University Hospital / ERIAS - Inserm U1219 BPH | France |
| Jean | B | Craig | PhD | craigje@musc.edu | Medical University of South Carolina | United States |
| Juan Luis |  | Cruz Bermúdez | PhD | juanluis.cruz@salud.madrid.org | Hospital Universitario 12 de Octubre, Madrid, Spain | Spain |
| Jaime |  | Cruz Rojo | MD | jaime.cruz@salud.madrid.org | Hospital Universitario 12 de Octubre, Madrid, Spain | Spain |
| Arianna |  | Dagliati | MS, PhD | arianna.dagliati@unipv.it | University of Pavia, Italy | Italy |
| Mohamad |  | Daniar | MSIS | mohamad.daniar@childrens.harvard.edu | Boston Children's Hospital | United States |
| Christel |  | Daniel |  | christel.daniel@aphp.fr | APHP Greater Paris University Hospital, INSERM | France |
| Anahita |  | Davoudi | PhD | anahita.davoudi@pennmedicine.upenn.edu | University of Pennsylvania Perelman School of Medicine | United States |
| Batsal |  | Devkota |  | devkotab@email.chop.edu | Department of Biomedical and Health Informatics, Children's Hospital of Philadelphia | United States |
| Julien |  | Dubiel |  |  | APHP Greater Paris University Hospital | France |
| Scott | L | DuVall |  | scott.duvall@va.gov | VA Informatics and Computing Infrastructure, VA Salt Lake City Health Care System | United States |
| Loic |  | Esteve |  |  | SED/SIERRA, Inria Centre de Paris | France |
| Shirley |  | Fan |  | shirlfan@med.umich.edu | University of Michigan | United States |
| Robert | W | Follett |  | rfollett@mednet.ucla.edu | Department of Medicine, David Geffen School of Medicine at UCLA | United States |
| Paula | S. A | Gaiolla |  | paulagaiolla@gmail.com | Internal Medicine Department of Botucatu Medical School, São Paulo State University, Botucatu, Brazil | Brazil |
| Thomas |  | Ganslandt | MD | thomas.ganslandt@medma.uni-heidelberg.de | University Medicine Mannheim, Heidelberg University | Germany |
| Noelia |  | García Barrio | MS | ngbarrio@salud.madrid.org | Hospital Universitario 12 de Octubre, Madrid, Spain | Spain |
| Nils |  | Gehlenborg |  | nils@hms.harvard.edu | Harvard Medical School | United States |
| Alon |  | Geva | MD, MPH | alon.geva@childrens.harvard.edu | Boston Children's Hospital, Boston, MA | United States |
| Tobias |  | Gradinger | MD, BSc | tobias.gradinger@medma.uni-heidelberg.de | University Medicine Mannheim, Heidelberg University | Germany |
| Alexandre |  | Gramfort |  | alexandre.gramfort@inria.fr | Université Paris-Saclay, Inria, CEA | France |
| Romain |  | Griffier |  | romain.griffier@chu-bordeaux.fr | Bordeaux University Hospital | France |
| Nicolas |  | Griffon |  | nicolas.griffon@aphp.fr | APHP Greater Paris University Hospital, INSERM | France |
| Olivier |  | Grisel |  |  | Université Paris-Saclay, Inria, CEA | France |
| Alba |  | Gutiérrez-Sacristán | PhD | alba_gutierrez@hms.harvard.edu | Harvard Medical School | United States |
| David | A | Hanauer | MD, MS | hanauer@umich.edu | University of of Michigan Medical School, Ann Arbor, Michigan, USA | United States |
| Christian |  | Haverkamp |  |  | Institute of Digitalization in Medicine, Faculty of Medicine and Medical Center, University of Freiburg, Germany | Germany |
| Darren | W | Henderson |  | darren.henderson@uky.edu | University of Kentucky | United States |
| Martin |  | Hilka |  |  | APHP Greater Paris University Hospital | France |
| John | H | Holmes | MS, PhD | jhholmes@pennmedicine.upenn.edu | University of Pennsylvania Perelman School of Medicine (inst), Philadelphia, Pennsylvania, United States of America | United States |
| Chuan |  | Hong | PhD | Chuan_Hong@hms.harvard.edu | Harvard Medical School | United States |
| Petar |  | Horki |  | horki@imbi.uni-freiburg.de | Institute of Medical Biometry and Statistics, Medical Center, University of Freiburg | Germany |
| Meghan | R | Hutch | BS | meghan.hutch@northwestern.edu | Northwestern University | United States |
| Richard | W | Issitt | DClinP | richard.issitt@gosh.nhs.uk | Great Ormond Street Hospital for Children, UK | United Kingdom |
| Anne Sophie |  | Jannot |  | annesophie.jannot@aphp.fr | Department of Biomedical Informatics, HEGP,  APHP Greater Paris University Hospital | France |
| Vianney |  | Jouhet | MD,PhD | vianney.jouhet@chu-bordeaux.fr | Bordeaux University Hospital | France |
| Mark | S | Keller |  | mark_keller@hms.harvard.edu | Harvard Medical School | United States |
| Katie |  | Kirchoff | MSHI | kirchoff@musc.edu | Medical University of South Carolina | United States |
| Jeffrey | G | Klann | MEng, PhD | jeff.klann@mgh.harvard.edu | Massachusetts General Hospital | United States |
| Isaac | S | Kohane | MD, PhD | isaac_kohane@harvard.edu | Harvard Medical School | United States |
| Ian | D | Krantz |  | krantz@email.chop.edu | Department of Pediatrics, Division of Human Genetcs, The Children's Hospital of Philadelphia and the Perelman School of Medicine at the University of Pennsylvania | United States |
| Detlef |  | Kraska | Dr. | detlef.kraska@uk-erlangen.de | University Hospital Erlangen | Germany |
| Ashok | K | Krishnamurthy | PhD | ashokk@email.unc.edu | University of North Carolina, Chapel Hill | United States |
| Sehi |  | L'Yi | PhD | sehi_lyi@hms.harvard.edu | Harvard Medical School | United States |
| Trang | T | Le | PhD | ttle@pennmedicine.upenn.edu | University of Pennsylvania Perelman School of Medicine | United States |
| Judith |  | Leblanc |  | judith.leblanc@aphp.fr | APHP Greater Paris University Hospital | France |
| Guillaume |  | Lemaitre |  |  | Université Paris-Saclay, Inria, CEA | France |
| Leslie |  | Lenert | MD, MS | lenert@musc.edu | Medical University of South Carolina | United States |
| Damien |  | Leprovost |  |  | Clevy.io | France |
| Molei |  | Liu | PhD | molei_liu@g.harvard.edu | Harvard T.H. Chan School of Public Health | United States |
| Ne Hooi Will |  | Loh | MBBS | will.loh@nus.edu.sg | National University Health System, Singapore | Singapore |
| Yuan |  | Luo | PhD | yuan.luo@northwestern.edu | Northwestern University | United States |
| Kristine | E | Lynch |  | kristine.lynch@va.gov | VA Informatics and Computing Infrastructure, VA Salt Lake City Health Care System | United States |
| Sadiqa |  | Mahmood |  | [sadiqa.mahmood@healthcatalyst.com](mailto:sadiqa.mahmood@healthcatalyst.com) | Health Catalyst, INC. | United States |
| Sarah |  | Maidlow |  | smaidlow@umich.edu | University of Michigan | United States |
| Alberto |  | Malovini | PhD | alberto.malovini@icsmaugeri.it | Istituti Clinici Scientifici Maugeri SpA SB IRCCS, Pavia, Italy. | Italy |
| Kenneth | D | Mandl | MD, MPH | kenneth_mandl@harvard.edu | Boston Children's Hospital | United States |
| Chengsheng |  | Mao | PhD | chengsheng.mao@northwestern.edu | Northwestern University | United States |
| Patricia |  | Martel |  | patricia.martel@aphp.fr | APHP Greater Paris University Hospital | France |
| Aaron | J | Masino | PhD | masinoa@email.chop.edu | Children's Hospital of Philadelphia | United States |
| Michael | E | Matheny |  | michael.matheny@va.gov | Tennessee Valley Healthcare System Veterans Affairs Medical Center | United States |
| Thomas |  | Maulhardt |  | thomas.maulhardt@uniklinik-freiburg.de | University Medical Center Freiburg, Germany | Germany |
| Maria |  | Mazzitelli | PhD | m.mazzitelli88@gmail.com | University Magna Graecia of Catanzaro, Italy | Italy |
| Michael | T | McDuffie |  | michael_mcduffie@hms.harvard.edu | Harvard Medical School | United States |
| Arthur |  | Mensch |  |  | ENS, PSL University | France |
| Marianna |  | Milano | PhD | m.milano@unicz.it | University Magna Graecia of Catanzaro, Italy | Italy |
| Marcos | F | Minicucci |  |  | Internal Medicine Department of Botucatu Medical School, São Paulo State University, Botucatu, Brazil | Brazil |
| Bertrand |  | Moal | PhD | bertrand.moal@chu-bordeaux.fr | Bordeaux University Hospital | France |
| Jason | H | Moore | PhD | jhmoore@upenn.edu | University of Pennsylvania Perelman School of Medicine | United States |
| Jeffrey | S | Morris |  | jeffrey.morris@pennmedicine.upenn.edu | University of Pennsylvania Perelman School of Medicine | United States |
| Michele |  | Morris | BA | mim18@pitt.edu | University of Pittsburgh | United States |
| Karyn | L | Moshal |  | karyn.moshal@gosh.nhs.uk | Great Ormond Street Hospital for Children, UK | United Kingdom |
| Sajad |  | Mousavi | PhD | sayedsajad_mousavi@hms.harvard.edu | Harvard Medical School | United States |
| Danielle | L | Mowery | PhD | dlmowery@pennmedicine.upenn.edu | University of Pennsylvania Perelman School of Medicine | United States |
| Douglas | A | Murad |  | dmurad@mednet.ucla.edu | David Geffen School of Medicine at UCLA | United States |
| Shawn | N | Murphy | MD, PhD | snmurphy@partners.org, googledoc is snmurphy00@gmail.com | Massachusetts General Hospital | United States |
| Thomas | P | Naughton | BA | thomas_naughton@hms.harvard.edu | Harvard Medical School | United States |
| Antoine |  | Neuraz | MD, PhD | antoine.neuraz@aphp.fr | Hôpital Necker-Enfants Malade, Assistance Publique Hôpitaux de Paris (APHP), University of Paris | France |
| Kee Yuan |  | Ngiam | MBBS, MRCS, MMed, FRCS | kee_yuan_ngiam@nuhs.edu.sg | National Univerisity Health Systems Singapore | Singapore |
| Jihad |  | Obeid | MD, FAMIA | jobeid@musc.edu | Medical University of South Carolina | United States |
| Marina | P | Okoshi | PhD | marina.okoshi@unesp.br | Internal Medicine Department of Botucatu Medical School, São Paulo State University, Botucatu, Brazil | Brazil |
| Karen | L | Olson | PhD | karen.olson@childrens.harvard.edu | Boston Children's Hospital and Harvard Medical School | United States |
| Gilbert | S. | Omenn | MD, PhD | gomenn@umich.edu | University of Michigan | United States |
| Nina |  | Orlova |  |  | APHP Greater Paris University Hospital | France |
| Brian | D | Ostasiewski |  | bostasie@wakehealth.edu | CTSI, WFBMI, Wake Forest School of Medicine | United States |
| Nicolas |  | Paris |  | nicolas.paris@aphp.fr | APHP Greater Paris University Hospital | France |
| Lav | P | Patel | MS | lpatel@kumc.edu | University Of Kansas Medical Center | United States |
| Miguel |  | Pedrera Jimenez | MS | miguel.pedrera@salud.madrid.org | Hospital Universitario 12 de Octubre, Madrid, Spain | Spain |
| Nathan | P | Palmer | PhD | nathan_palmer@hms.harvard.edu | Harvard Medical School | United States |
| Danielle |  | Pillion | MS | danielle_pillion@hms.harvard.edu | Harvard Medical School | United States |
| Hans | U | Prokosch |  | ulli.prokosch@uk-erlangen.de | University of Erlangen-Nürnberg | Germany |
| Robson | A | Prudente | PhD | robsonapp@gmail.com | Clinical Research Unit São Paulo State University, Brazil | Brazil |
| Víctor |  | Quirós González | MS | victor.quiros@salud.madrid.org | Hospital Universitario 12 de Octubre, Madrid, Spain | Spain |
| Rachel | B | Ramoni |  | rachel.ramoni@va.gov | Office of Research and Development, Department of Veterans Affairs | United States |
| Maryna |  | Raskin |  | maryna.raskin@healthcatalyst.com | Health Catalyst, INC. | United States |
| Siegbert |  | Rieg |  | siegbert.rieg@uniklinik-freiburg.de | Division of Infectious Diseases, Department of Medicine II, Medical Center – University of Freiburg, Faculty of Medicine | Germany |
| Gustavo |  | Roig Domínguez | MS | gustavo.roig@h12o.es | Hospital Universitario 12 de Octubre, Madrid, Spain | Spain |
| Elisa |  | Salamanca |  | e_l_salamanca@yahoo.fr | APHP Greater Paris University Hospital | France |
| Malarkodi | J | Samayamuthu | MD | mas609@pitt.edu | University of Pittsburgh | United States |
| Arnaud |  | Sandrin |  | arnaud.sandrin@aphp.fr | APHP Greater Paris University Hospital | France |
| Maria |  | Savino | MS | [maria1_savino@asst-pavia.it](mailto:maria1_savino@asst-pavia.it) | Management Engineer, Direction | Italy |
| Emily | R | Schriver | MS | emily.schriver@pennmedicine.upenn.edu | University of Pennsylvania Health System | United States |
| Juergen |  | Schuettler |  | juergen.schuettler@kfa.imed.uni-erlangen.de | Department of Anesthesiology, University Hospital Erlangen, FAU Erlangen-Nürnberg, Germany | Germany |
| Luigia |  | Scudeller |  | luigia.scudeller@policlinico.mi.it | Scientific Direction, IRCCS Ca' Granda Ospedale Maggiore Policlinico di Milano | Italy |
| Neil | J | Sebire | MD, FRCPath | neil.sebire@gosh.nhs.uk | Great Ormond Street Hospital for Children NIHR BRC, UK | United Kingdom |
| Pablo |  | Serrano Balazote | MD,MS | pserranob@salud.madrid.org | Hospital Universitario 12 de Octubre, Madrid, Spain | Spain |
| Patricia |  | Serre |  |  | APHP Greater Paris University Hospital | France |
| Arnaud |  | Serret-Larmande | MD | arnaud.serret-larmande@aphp.fr |  | France |
| Domenick |  | Silvio |  | silviod@umich.edu | University of Michigan, MICHR | United States |
| Piotr |  | Sliz |  | piotr.sliz@childrens.harvard.edu | Boston Children's Hospital | United States |
| Jiyeon |  | Son | MD | sonj@upmc.edu | University of Pittsburgh Medical Center | United States |
| Charles |  | Sonday |  | Charles.Sonday@sluhn.org | Critical Care Medicine, Department of Medicine, St. Luke's University Health Network, Bethlehm PA | United States |
| Andrew | M | South | MD, MS | asouth@wakehealth.edu | Brenner Children's Hospital, Wake Forest School of Medicine | United States |
| Anastasia |  | Spiridou | PhD, MSc | natassa.spiridou@gosh.nhs.uk | Great Ormond Street Hospital for Children, UK | United Kingdom |
| Amelia | LM | Tan | BSc, PhD | amelia_tan@hms.harvard.edu | Harvard Medical School | United States |
| Bryce | W.Q. | Tan | MBBS | bryce_tan@nuhs.edu.sg | National University Hospital, Singapore | Singapore |
| Byorn | W.L. | Tan | MBBS | byorn_wl_tan@nuhs.edu.sg | National University Hospital, Singapore | Singapore |
| Suzana | E | Tanni |  | suzanapneumo@gmail.com | Internal Medicine Department of Botucatu Medical School, São Paulo State University, Botucatu, Brazil | Brazil |
| Deanne | M | Taylor | PhD | taylordm@email.chop.edu | The Children's Hospital of Philadelphia and the University of Pennsylvania Perelman Medical School | United States |
| Ana | I | Terriza Torres | MS | anaisabel.terriza@salud.madrid.org | Hospital Universitario 12 de Octubre, Madrid, Spain | Spain |
| Valentina |  | Tibollo |  | valentina.tibollo@icsmaugeri.it | Laboratory of Informatics and Systems Engineering for Clinical Research, Istituti Clinici Scientifici Maugeri SpA SB IRCCS, Pavia, Italy. | Italy |
| Patric |  | Tippmann | MSc | tippmann@imbi.uni-freiburg.de | Institute of Medical Biometry and Statistics, Medical Center, University of Freiburg | Germany |
| Carlo |  | Torti | PhD | torti@unicz.it | University Magna Graecia of Catanzaro, Italy | Italy |
| Enrico | M | Trecarichi | PhD | em.trecarichi@unicz.it | University Magna Graecia of Catanzaro, Italy | Italy |
| Andrew | K | Vallejos |  | avallejo@mcw.edu | Clinical & Translational Science Institute, Medical College of Wisconsin | United States |
| Gael |  | Varoquaux |  |  | Université Paris-Saclay, Inria, CEA, Montréal Neurological Institute, McGill University | France |
| Jill-Jênn |  | Vie |  |  | SequeL, Inria Lille | France |
| Shyam |  | Visweswaran | MD, PhD | shv3@pitt.edu | University of Pittsburgh | United States |
| Michele |  | Vitacca | MD, PhD | michele.vitacca@icsmaugeri.it | ICS S. Maugeri IRCCS Pavia Italy | ITALY |
| Kavishwar | B | Wagholikar | MBBS, PhD | kwagholikar@mgh.harvard.edu | Department of Medicine, Massachusetts General Hospital, Boston, USA | United States |
| Lemuel | R | Waitman |  | russ.waitman@health.missouri.edu | University of Missouri, Columbia. MO | United States |
| Demian |  | Wassermann |  |  | Université Paris-Saclay, Inria, CEA | France |
| Griffin | M | Weber |  | weber@hms.harvard.edu | Harvard Medical School | United States |
| Yuan |  | William |  | william_yuan@g.harvard.edu | Harvard Medical School | United States |
| Zongqi |  | Xia | MD, PhD | zxia1@post.harvard.edu | University of Pittsburgh | United States |
| Nadir |  | Yehya | MD, MSCE | yehyan@email.chop.edu | Children's Hospial of Philadelphia and University of Pennsylvania | United States |
| Alberto |  | Zambelli |  | alberto.zambelli@asst-pg23.it | ASST Papa Giovanni XXIII, Bergamo | Italy |
| Harrison | G | Zhang |  | harrison_zhang@hms.harvard.edu | Harvard Medical School | United States |
| Chiara |  | Zucco | PhD | chiara.zucco@unicz.it | University Magna Graecia of Catanzaro, Italy | Italy |
| James | B | Norman |  | james_norman@hms.harvard.edu | Harvard Medical School | United States |
| Anupama |  | Maram | MS | anupama_maram@hms.harvard.edu | Harvard Medical School | United States |
| Nicholas | W | Brown | MEng | nicholas_brown@hms.harvard.edu | Harvard Medical School | United States |
| Kenneth | M | Huling | HS | kenneth.huling@gmail.com | Harvard Medical School | United States |
| Emily | R | Pfaff | PhD | epfaff@email.unc.edu | UNC Chapel Hill | United States |
| Lorenzo |  | Chiudinelli | PhD | chiudinelli.lorenzo@gmail.com | ASST Papa Giovanni XXIII, Bergamo | Italy |
| Yi-Ju |  | Tseng | PhD | yjtseng.info@gmail.com | Chang Gung University | Taiwan |

**Supplementary Table 2**: Participating sites.


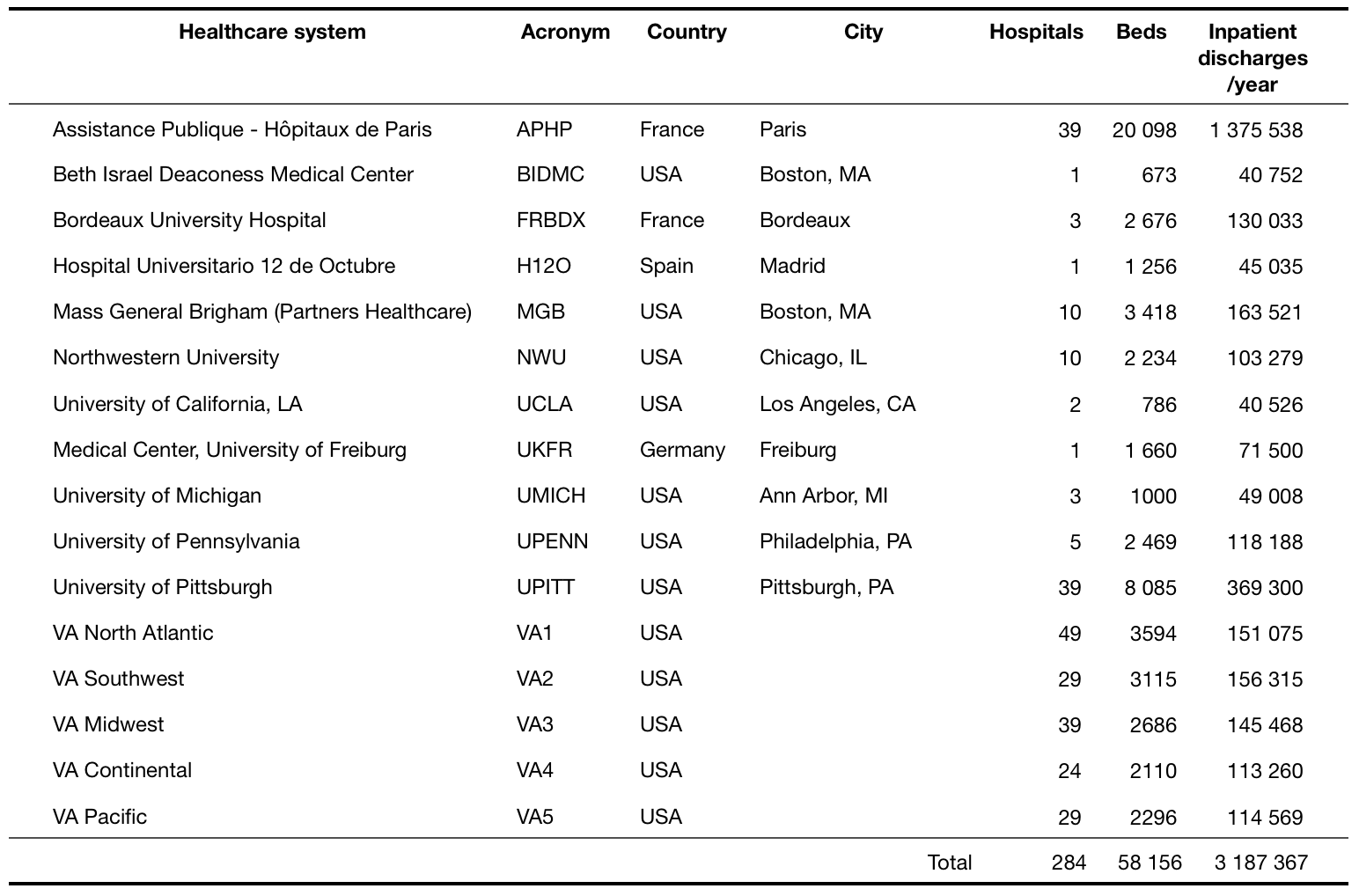


**Supplementary Table 3**: Three comma-separated values (CSV) files and their descriptions. Sites optionally obfuscated the values in any of these files by replacing small counts with “-99.” Sites indicated missing data or data that they were unable to obtain (e.g., race and ethnicity not collected at some sites) with “-999.”

| **Data File** | **Description** |
| --- | --- |
| LocalClinicalCourse.csv | This file has one row per patient and "days_since_admission". It indicates which days the patient was in the hospital and when the patient became severe or died |
| LocalObservations.csv | This file has one row per patient, days_since_admission, and "concept". Concepts include (1) first three character ICD9/10 diagnoses, (2) Phase 1.1 medication classes, (3) Phase 1.1 LOINC labs, and (4) ICD9/10 procedure codes used in Phase 1.1 to determine severity. Diagnoses and medications start at day -365 (one year before the patient was admitted) and go through the current date. Lab test results start at day -60 (two months before the patient was admitted) and go through the current date. Procedure codes start at day 0 (the day of admission) and go through the current date. Lab concepts have a numeric value. For other concepts, the value is "-999". If a patient has the same lab repeated in the same day, then the value in this CSV file is the mean of all the test results from that day. Note that this files does not contain every observation that a patient has. It only contains the rolled-up concepts that were quality checked in Phase 1.1. |
| LocalPatientSummary.csv | This file has one row per patient.  It contains the initial COVID inpatient admission date, the date patients became severe (if applicable), and the date they died (if applicable).  A "still_in_hospital" flag indicates if the patient was still in the hospital when the script was run, which means the final outcome (discharge vs death) is not yet known.  It also includes sex, age_group, and race.  A "race_collected" flag indicates whether the site capture race information. If race_collected=0 (no), then race is set to "other". |

**Supplementary Table 4**: AUCs of cox regression model across different sites, countries, and continents.

| **site.from/to** | **all** | **France** | **Germany** | **SITE1** | **SITE10** | **SITE11** | **SITE12** | **SITE13** | **SITE14** | **SITE15** | **SITE16** | **SITE2** | **SITE3** | **SITE4** | **SITE5** | **SITE6** | **SITE7** | **SITE8** | **SITE9** | **Spain** | **US** |
| --- | --- | --- | --- | --- | --- | --- | --- | --- | --- | --- | --- | --- | --- | --- | --- | --- | --- | --- | --- | --- | --- |
| **SITE1** | 0.82 | 0.82 | 0.84 | 0.81 | 0.84 | 0.80 | 0.81 | 0.82 | 0.88 | 0.88 | 0.81 | 0.85 | 0.84 | 0.80 | 0.81 | 0.84 | 0.75 | 0.79 | 0.83 | 0.81 | 0.83 |
| **SITE10** | 0.83 | 0.84 | 0.79 | 0.81 | 0.85 | 0.81 | 0.85 | 0.83 | 0.90 | 0.91 | 0.79 | 0.88 | 0.79 | 0.81 | 0.83 | 0.84 | 0.76 | 0.81 | 0.85 | 0.79 | 0.84 |
| **SITE11** | 0.82 | 0.83 | 0.74 | 0.80 | 0.86 | 0.79 | 0.83 | 0.82 | 0.87 | 0.87 | 0.79 | 0.87 | 0.74 | 0.80 | 0.80 | 0.83 | 0.76 | 0.81 | 0.84 | 0.79 | 0.83 |
| **SITE12** | 0.82 | 0.83 | 0.83 | 0.80 | 0.85 | 0.78 | 0.81 | 0.82 | 0.91 | 0.87 | 0.80 | 0.86 | 0.83 | 0.83 | 0.82 | 0.84 | 0.78 | 0.80 | 0.84 | 0.80 | 0.83 |
| **SITE13** | 0.81 | 0.83 | 0.85 | 0.81 | 0.84 | 0.78 | 0.80 | 0.73 | 0.90 | 0.88 | 0.81 | 0.86 | 0.85 | 0.79 | 0.79 | 0.82 | 0.75 | 0.81 | 0.82 | 0.81 | 0.81 |
| **SITE14** | 0.82 | 0.80 | 0.81 | 0.78 | 0.85 | 0.76 | 0.83 | 0.82 | 0.84 | 0.87 | 0.79 | 0.83 | 0.81 | 0.80 | 0.84 | 0.84 | 0.79 | 0.80 | 0.81 | 0.79 | 0.83 |
| **SITE15** | 0.79 | 0.80 | 0.86 | 0.78 | 0.79 | 0.75 | 0.82 | 0.81 | 0.84 | 0.54 | 0.78 | 0.84 | 0.86 | 0.75 | 0.80 | 0.81 | 0.75 | 0.77 | 0.76 | 0.78 | 0.78 |
| **SITE2** | 0.81 | 0.76 | 0.77 | 0.79 | 0.85 | 0.80 | 0.83 | 0.81 | 0.90 | 0.86 | 0.79 | 0.72 | 0.77 | 0.81 | 0.81 | 0.83 | 0.75 | 0.77 | 0.83 | 0.79 | 0.83 |
| **SITE3** | 0.75 | 0.73 | 0.79 | 0.73 | 0.75 | 0.69 | 0.76 | 0.74 | 0.81 | 0.85 | 0.74 | 0.70 | 0.79 | 0.68 | 0.79 | 0.76 | 0.71 | 0.77 | 0.73 | 0.74 | 0.75 |
| **SITE4** | 0.83 | 0.83 | 0.86 | 0.81 | 0.83 | 0.76 | 0.82 | 0.83 | 0.90 | 0.91 | 0.80 | 0.86 | 0.86 | 0.73 | 0.82 | 0.84 | 0.78 | 0.81 | 0.83 | 0.80 | 0.83 |
| **SITE5** | 0.82 | 0.82 | 0.81 | 0.80 | 0.83 | 0.78 | 0.81 | 0.81 | 0.88 | 0.89 | 0.79 | 0.86 | 0.81 | 0.82 | 0.84 | 0.85 | 0.76 | 0.79 | 0.84 | 0.79 | 0.83 |
| **SITE6** | 0.83 | 0.83 | 0.84 | 0.81 | 0.85 | 0.81 | 0.83 | 0.83 | 0.90 | 0.90 | 0.80 | 0.86 | 0.84 | 0.81 | 0.82 | 0.85 | 0.76 | 0.78 | 0.84 | 0.80 | 0.84 |
| **SITE7** | 0.81 | 0.82 | 0.84 | 0.80 | 0.85 | 0.79 | 0.84 | 0.83 | 0.88 | 0.90 | 0.79 | 0.85 | 0.84 | 0.76 | 0.82 | 0.82 | 0.72 | 0.79 | 0.82 | 0.79 | 0.82 |
| **SITE8** | 0.81 | 0.82 | 0.82 | 0.81 | 0.84 | 0.79 | 0.81 | 0.82 | 0.87 | 0.87 | 0.80 | 0.85 | 0.82 | 0.76 | 0.80 | 0.81 | 0.78 | 0.77 | 0.80 | 0.80 | 0.81 |
| **SITE9** | 0.82 | 0.83 | 0.82 | 0.81 | 0.85 | 0.80 | 0.83 | 0.82 | 0.88 | 0.88 | 0.80 | 0.86 | 0.82 | 0.82 | 0.81 | 0.84 | 0.76 | 0.79 | 0.83 | 0.80 | 0.83 |
